# Reusable Extractant and Direct Catalytic Mediation of Water/Oil/Chlorodifluoromethane Nano-Emulsion in Natural Gas Condensate for Efficient Conversion of Chloride Impurities Into the Dicopper Chloride Trihydroxide Nanoparticles

**DOI:** 10.3389/fchem.2022.823357

**Published:** 2022-04-26

**Authors:** Zohre Banan Khorshid, Mohammad Mahdi Doroodmand

**Affiliations:** Department of Chemistry, College of Sciences, Shiraz University, Shiraz, Iran

**Keywords:** natural gas condensate, mercury and chloride impurities, nano-emulsion, dicopper chloride trihydroxide, catalytic mediator, agricultural pesticide

## Abstract

This research introduces an oil-in-water (O/W) nano-emulsion (oil-water- ${\text{CHClF}}_{2}$ ) as the reusable extractant phase using liquid–liquid extraction methodology for the removal efficiency of Cl^−^ and Hg(0) [between 90% and ∼100%, deepening on the nature of the natural gas condensate (NGC)] at a brief separation time (<3.0 min). The achieved safety of the NGC using this nano-emulsion results in efficient reduction in the corrosion rate during testing iron-based fragments (vs. the untreated ones as controls) and increase in the NGC economic value. Another advantage of the synthesized nano-emulsion is its capability and catalytic mediating behavior to efficiently separate and synthesize highly pure dicopper chloride trihydroxide nanoparticles. The synthesized nanoparticles were characterized by different analytical methods such as Fourier transform infrared spectrometry, X-ray diffraction, X-ray photoelectron spectrometry, and direct visualization by some electron microscopies. Direct synthesis, fast synthetic time (<3.0 min), high purity (>99%), and scalability are the main advantages of this synthetic method. This nanoparticle is not only safe but also is efficiently applicable in different industries, especially as an eco-friendly agricultural pesticide for different plants and tress such as pistachio. Consequently, this method is accepted as direct, simple, low-cost, and scalable conversion of some upstream industries with the downstream ones. All these possibilities are attributed to the intermediate transport properties of the introduced O/W nano-emulsion. At this condition, this reagent plays role as a recycled motor for the NGC purification and conversion of these impurities into the safe and usable products. To the best of knowledge, this research is considered as the first report that shows application of this *O/W* medium for both chloride and mercury removal from the NGC and its direct use as top element in the synthesis of eco-friendly nanoparticles. This system is applicable in some parts of the fuel and oil centers of the “Middle East.”

## Introduction

Natural gas condensates (*NGCs*) often involve serious impurities such as chloride ion $\left(Cl^{-}\right)$ , hydrogen sulfide (*H*_*2*_ *S*), mercaptans (*R-SH*), mercury element [Hg(0)], and inorganic salts. These impurities are often attributed to the neighborhood of the NGCs to the geological contexts (Sabbaghi et al., 2012). However, in the NGCs, some pollutions, especially ${\text{Cl}}^{-}$ and Hg(0), are so high that they can even be detected *via* direct visualizing their intensive colorimetric turbidity in some parts of the fuel and oil centers, especially in the “Middle East” (Pierce et al., 2007; Sabbaghi et al., 2012; Hou et al., 2013; Wu et al., 2013; Lavela et al., 2015; Sun et al., 2019).

In fact, the ${\text{Cl}}^{-}$ , as one of the most aggressive ionic species, can seriously cause inadequate flammability and pitting corrosion (Ergun and Turan, 1991); this issue would consequently produce failure of the oil/gas production lines and the transportation equipment (Jiang et al., 2013). The importance of this problem is more serious when some different metal-chloride complexes are formed that play significant role as catalytic poisoning (Argyle and Bartholomew, 2015). Besides, elemental mercury [Hg(0)], even at its trace levels (Bingham, 1990), has also corrosive property that extremely damages some piping equipment, especially the aluminum heat exchangers (Wilhelm, 2009). Moreover, Hg(0) extremely provides health problems for the industrial workers (Wilhelm and Bloom, 2000). All these problems reveal strong demand for purification (removal) of the NGC from these serious impurities.

Although different methodologies have been proposed for the ${\text{Cl}}^{-}$ removal from various real samples (Pierce et al., 2007; Sabbaghi et al., 2012; Hou et al., 2013; Wu et al., 2013; Lavela et al., 2015; Sun et al., 2019), but (to the best of knowledge) these methods have not been suitable, especially because of altered problems such as sophisticated matrix and irreversible fouling effect(s) of the NGC. On the other hand, other systems such as sorbent-based mercuric filters (Yan, 1987; Yang et al., 2007; Abai et al., 2015; Liu et al., 2020) have frequently high cost, small efficiency, and often need high temperature. Hence, current study introduces a nano-emulsion as extractant phase (extraction solvent) for efficient and simultaneous removal of the ${\text{Cl}}^{-}$ and Hg(0) using liquid–liquid extraction (*LLE*) methodology. In addition, the nano-emulsion’s catalytic mediation results in the conversion of the extracted ${\text{Cl}}^{-}$ to the dicopper chloride trihydroxide $\left({\text{Cu}}_{2}{\left(\text{OH}\right)}_{3}{\text{Cl}}_{\left(\text{s}\right)}\right)$ nanoparticles (Jackson and Fulton, 1996; Scott, 2000; Lubej et al., 2004; Luo et al., 2005; Lu et al., 2010) with eco-friendly agricultural pesticide applications.

Briefly, dicopper chloride trihydroxide is considered as an appropriate compound that is useful as an effective pesticide for various plants and trees, especially pistachio (Brühl et al., 2021). The general synthetic process of this compound is often based on the reaction between copper salts and chloride compounds at the basic condition (Jackson and Fulton, 1996; Lubej et al., 2004). However, the efficiency of the formation of this compound is seriously limited due to some different side reactions. The attribution of these reactions leads to the formation of some impurities such as Cu(OH)_2_ and CuCl_2_, besides some various coordination compounds such as CuCl_3_^−^ and CuCl_4_^2−^. (Scott, 2000; Hussain et al., 2019; FAO/WHO, 2021). These producers are often generated on the basis of different mechanisms such as coprecipitation and crystal-based complex formation.

Besides the abovementioned problems, the lack control on other factors like size distribution and the morphology is also other problems, owing to the harness of the control of the relative supper saturation and the absence of any suitable template and capping agents (Luo et al., 2005; Lu et al., 2010; Chen and Zhong, 2022; Pathania et al., 2022). Fortunately, the introduced oil-in-water (O/W) emulsion plays significant roles as almost all of the abovementioned reagents for reaching to pure, size-controllable, and high-efficient pesticide. Therefore, the novelty of this study is brief: Not only is the Cl^−^ and Hg(0) removal from the NGC but also is accessing an important and applicable compound. To the best of knowledge, no applicable methodology has been introduced for solving the current problem, especially at the industrial scale.

## Experimental

### Materials

The necessity reagents related to the nano-emulsion syntheses have been from Merck Company; their information and the NGC sample data were given in Supplementary Material S2.1. How to prepare this nano-emulsion as an *O/W* medium was presented in detail in Supplementary Material S2.2. Stable homogeneity (Chen and Zhong, 2022; Pathania et al., 2022) of the synthesized O/W nano-emulsion was confirmed for a long time (up to 24 h), which could be re-homogenized simply *via* vigorously shaking for a few seconds (maximum 10 s).

### Nano-Emulsion Preparation (Standard Extracting Medium)

The *HCFC-22 (R*_*22*_ *)–*treated oil was prepared using descriptions methodology in the previous study (Mohammadi et al., 2016; Banan Khorshid et al., 2021). Briefly, the stock nano-emulsion medium with oil concentration of 20.80 g L^−1^, as the extracting medium, was simply prepared by weighting 1,040.0 mg of this oil sample. Then, it was mixed with the triply distilled water (20.0 ml) during stirring using a magnet stirrer for a few minutes (3.0 min) at 400 ± 2 rpm using a hotplate stirrer (RT_2_, Denville Scientific Inc., American Cleanstat, LLC, Phenix Research Products Inc., Candler, NC, USA). In this synthetic process, the stability of the R_22_-treated oil for a long time. The results exhibited no significant desorption of the R_22_ from the oil matrix. This practically revealed no toxicity of the R_22_-treated oil using this procedure.

The R_22_ was then purged with flow rate of 5.00 ± 0.01 ml min^−1^ for 10.0 min using a mass flow controller (MFC, Bronkhorst high-tech. B.V., England). After that, it was transferred into a 50.0-ml glass volumetric flask (Thermo Scientific^™^ Nalgene^™^ Class B Polypropylene Copolymer Volumetric Flasks with Closure) and diluted to the mark using the triply distilled water. Then, it was vigorously shaken for about 1 min to form the homogenous nano-emulsion. The synthesized nano-emulsion was visualized using a fluorescence optical microscope (Olympus IX70, UK).

Daily dilution of the stock nano-emulsion was achieved *via* sampling a fixed volume of the stock solution using an “*Eppendorf*” pipette (Eppendorf^®^ Research^®^ Plus Pipettes; Sigma-Aldrich) and transferring into a glass volumetric flask. Afterward, it was stirred for 1 min and sonicated in a sonication bath (Ultrasonic cleaner, 400 W, 9.5 L, 40 kHz, C250X, MEGA Lab, UK) for 30.0 s at room temperature. Finally, it was diluted to the mark using the triply distilled water. As is expected, direct imaging the nano-emulsion, multiple circular phases revealed the existence of plenty of efficient interfaces for direct interaction with the NGC sample during simultaneous ${\text{Cl}}^{-}$ and the Hg(0) removal purpose.

It also should be noted that the concentration of the nano-emulsion was expressed in grams per liter. This was because of *1*) better dealing with the R_22_ gas, *2*) dependency of the concentration to the temperature, and *3*) the lack of knowledge about the precise gram formula weights of each oil and the NGC. Consequently, it was impossible to introduce the composite of the synthesized nano-emulsion based on mole fraction. Therefore, in this experiment, it was decided to focus on some general terms such as flow rate, volume, and purging time for the reproducible synthesis of the nano-emulsions. Nevertheless, about other reagents such as $Ag^{+}$ and $Cu^{2+}$ solutions, the concentrations were reported based on moles per liter to have better comparison and interpretation during the optimization process.

### Liquid–Liquid Extraction

Complete detail of the LLE method is given in Supplementary Material S2.3.

### Analysis Process

Experimental detail about the semi-quantitative test for the ${\text{Cl}}^{-}$ detection *via* silver chloride (AgCl) particles formation has been reported in Supplementary Material S2.4. In addition, the corrosion test was evaluated *via* following the weight loss and direct visualization of the corrosion of some iron fragments during contacting with the treated and untreated NGCs, under similar conditions, by scanning electron microscopy (SEM) as shown in Supplementary Material S2.5. Besides, quantitative tests for the Hg(0) determination based on standard addition and external calibration methods using ion-exchange chromatography (*IEC*) and cold vapor–atomic absorption spectrometry (*CV-AAS*) were shown in Supplementary Material S2.6.

### Formation of Dicopper Chloride Trihydroxide Nanoparticles

Along the accomplishing the extraction step, ${\text{Cu}}_{2}{\left(\text{OH}\right)}_{3}{\text{Cl}}_{\left(\text{s}\right)}$ nanoparticles were generated based on the catalytic mediation of the nano-emulsion using the ${\text{Cu}}^{2+}$ solution as both the de-emulsifier and the responsible reagent according to the recommended procedure (see Supplementary Material S2.7). In particular, the term “*Catalytic Mediation*” is a famous phrase that can be looked up in different reported articles (D’Acunzo and Galli, 2003; D'Alfonso et al., 2014). In this research, the synthesized nano-emulsion was therefore applied to the system to finalize the precipitation process and removal of insoluble particles with maximum removal efficiency.

## Results and Discussion

### Evidence About the Morphology of the Synthetized Nano-Emulsion

In the synthesized nano-emulsions, often, these compounds are evaluated indirectly *via* following their chemical stability. In this nano-emulsion, because of its Brownian motion, besides its thermal stability, often, it is impossible to directly observe them even by cryo–transmission electron microscopy (TEM) imaging. Hence, in the synthesized nano-emulsion, the characterization was limited only to the evaluation of its mechanical stability for a long time, for instance, 24 h, which could be re-homogenized simply *via* vigorously shaking for a few seconds (maximum 10 s). This characterization was considered as appropriate and acceptable methodology for selection of the term “*Nano-Emulsion*” about this system (Chen and Zhong, 2022; Pathania et al., 2022).

### Chloride and Mercury Removal From the NGC

The ${\text{Cl}}^{-}$ and Hg(0) impurities removal process from sophisticated organic phases such as NGC and their determining methodology have regarded with different problems (see Supplementary Material S3.1). These challenges therefore encouraged us to focus on the LLE as a simple method. This process was also along with adopting direct observing (visualization) probe for semi-quantitative tracing the optimization steps during introduction of the synthetic nano-emulsion. Formation of inorganic particles therefore revealed the presence of the ${\text{Cl}}^{-}$ as corrosive impurity into the NGC samples.

During the optimization process (see Supplementary Materials S3.2 and S3.3), maximum insoluble inorganic particles (colloids) were observed, when using the ${\text{ Ag}}^{+}$ solution, compared to other cationic species (Table 1, see also Supporting Information, Supplementary Figure S1), with optimum concentration as large as 0.2350 mol L^−1^ (see Supporting Information, Supplementary Material S3.3).

**TABLE 1:** Effect of nano-emulsion (oil, 5,200.0 μg ml^−1^) containing different cations on chloride removal from the NGC sample at 25°C.

Extracting medium	Chloride concentration (µg ml^−1^) in NGC ± SD ^a^	Extraction efficiency (%)
	(*n* = 3) ^b^	
Before extraction	315.0 ± 4.0	—
Ag^+^	23.0 ± 2.0	92.7
Cu^2+^	21.0 ± 1.0	93.3
Fe^3+^	235.0 ± 4.0	25.1
Fe^2+^	220.0 ± 2.0	30.1
Mn^2+^	205.0 ± 3.0	34.9
Ca^2+^	230.0 ± 3.0	27.0

a SD, standard deviation.

b *n* = number of replicate analyses.

On the basis of the results (Table 1), only the nano-emulsion, containing the ${\text{Cu}}^{2+}$ , had good effect on the ${\text{Cl}}^{-}$ removal from the NGC matrix. Whereas, in other cations problems, such as *1*) nano size of the generated particles, *2*) necessity to introduce excess amounts of cation, *3*) long extraction time, *4*) hard conditions (such as necessity of the suspension to a shaker for long time, *5*) high cost, and/or *6*) template effects of the nano-emulsion, extremely limited the extraction process. To select the ${\text{Cu}}^{2+}$ solution, its nitrate salt was considered as the best reagent because of its pitting corrosion inhibition property (Ergun and Turan, 1991). On the basis of the analysis, this process therefore led to efficiently removal of both ${\text{Cl}}^{-}$ and Hg(0) from the NGC matrix. As conclusion, for the efficient extraction and effective mass transfer of the ${\text{Cl}}^{-}$ from the NGC samples to the aqueous phase through using the nano-emulsion, Cu^2+^ played role as both extracting and de-emulsifying agents.

### Optimization Process

Detailed study about the optimization of effective factors by on one-factor-at-a-time method such as ${\text{Cu}}^{2+}$ concentration, oil volume, and effect(s) of acidity/basicity condition (pH value) was reported quantitatively in Table 2 and evaluated by photographic imaging (see Supplementary Materials S3.4–S3.6 and Supplementary Figures S3 and S4).

**TABLE 2:** Effect of Cu^2+^ concentration in nano-emulsion (oil, 5,200.0 μg ml^−1^) on chloride extraction efficiency.

Cu^2+^ concentration (mol L^−1^) (apparent concentration)	Chloride concentration (µg ml^−1^) in NGC^a^ (after extraction) ± SD ^b^	Extraction efficiency (%)
	(*n* = 3)^c^	
3.0 × 10^−3^	219 ± 1	30.5
6.6 × 10^−3^	211 ± 2	33.0
1.3 × 10^−2^	112 ± 3	64.0
1.7 × 10^−2^	52 ± 4	83.5
2.0 × 10^−2^	32 ± 3	89.8
2.3 × 10^−2^	29 ± 4	90.8
2.6 × 10^−2^	27 ± 2	91.4

a Chloride concentration before extraction was 315 ± 4 (*n* = 3).

b SD, standard deviation.

c *n* = number of replicate.

On the basis of the results (Table 2), concatenation of Cu^2+^ was strongly dependent on the oil concentration. In another word, optimum concentration of oil simply led to decrease the amount of Cu^2+^ that was needed for playing role as both complex forming and de-emulsifying agent. This condition not only did not provide major change(s) in the matrix of the NGC sample during the extraction process but also majorly reduced the probable side effect of residual Cu^2+^ medium, possibly present inside the NGC after applying the extraction process. Therefore, it was needed to have full confidence about the optimum Cu^2+^ concentration in the presence of a fixed amount of oil solution using another independent analysis such as direct visualization of the AgCl particles formations (Hong et al., 2006) inside the NGC after introducing different amounts of Cu^2+^.

**FIGURE 1:**
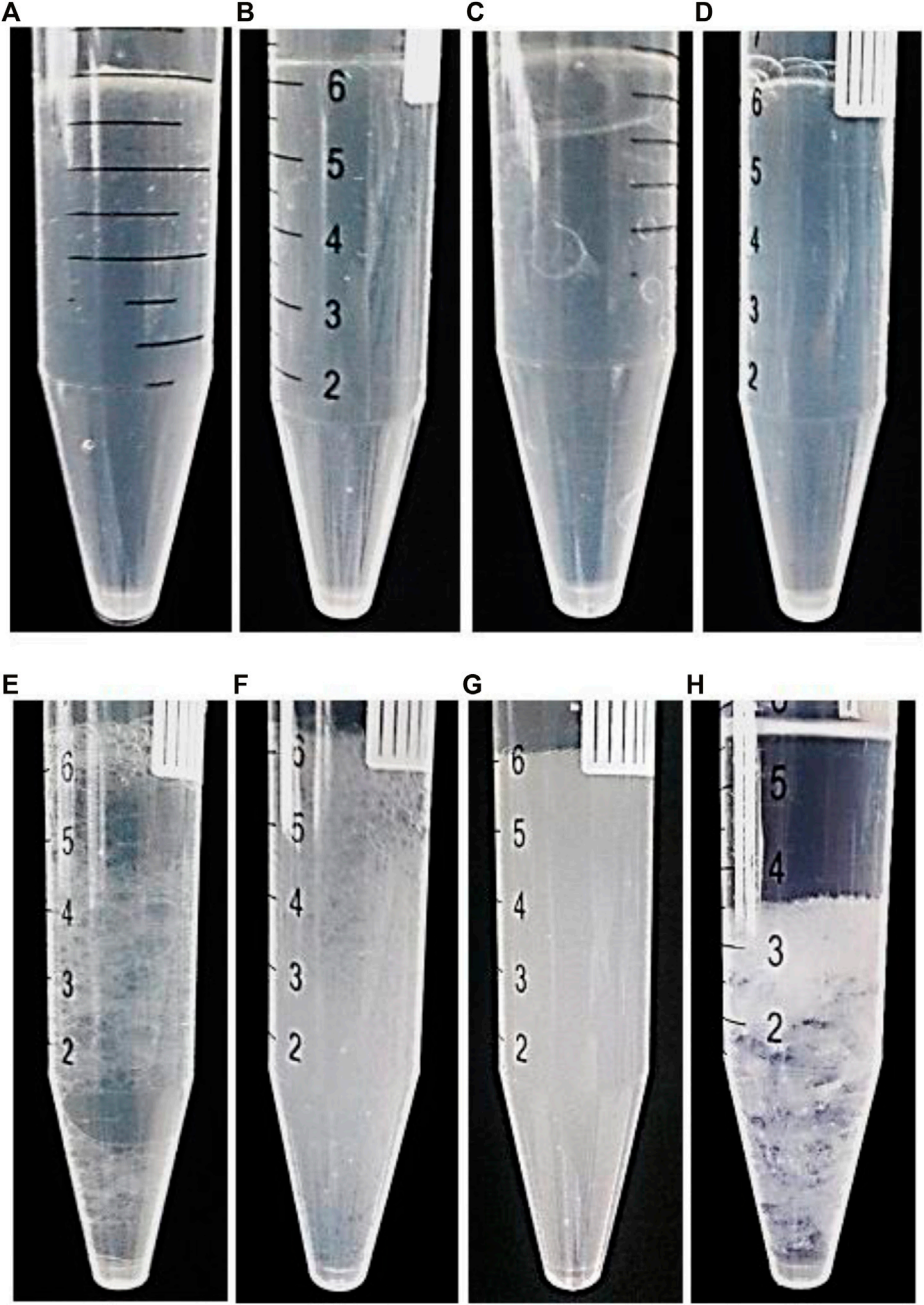
Re-optimization of Cu^2+^ concentration; AgCl formation after extraction by nano-emulsion in same oil concentration (10,400.0 μg ml^−1^) and different Cu^2+^ concentrations (apparent concentration) of **(A)** 2.0 × 10^−2^, **(B)** 1.3 × 10^−2^, **(C)** 9.9 × 10^−3^, **(D)** 6.6 × 10^−3^, **(E)** 3.3 × 10^−3^, **(F)** 2.0 × 10^−3^, **(G)** 1.0 × 10^−3^, and **(H)** 0.0 mol L^−1^.

For this purpose, six nano-emulsion samples were prepared with the same oil concentration (oil, 10,400.0 μg ml^−1^; which was optimized in the previous section) in the presence of different Cu^2+^ concentrations (lower concentrations): 1.3 × 10^−2^, 9.9 × 10^−3^, 6.6 × 10^−3^, 3.3 × 10^−3^, 2.0 × 10^−3^, and 1.0 × 10^−3^ mol L^−1^ (apparent concentration). After performing the extraction process between 5.0 ml of NGC and the same volume according to the recommended procedure, the quantity of the generated AgCl particles was evaluated precisely according to the image photographs shown in Figure 1.

On the basis of the results (Figure 1), Cu^2+^ (6.6 × 10^−3^ mol L^−1^ ; apparent concentration) and oil (10,400.0 μg ml^−1^) showed acceptable effect with chloride removal efficiency to around 100% (only by a single step). Therefore, this value was selected as optimum Cu^2+^ concentration.

### Ionic Strength in Extractant Nano-Emulsion

Utilizing Cu(NO_3_)_2_ (6.6 × 10^−3^ mol L^−1^; apparent concentration) in extractant medium resulted in having ionic strength as large as around 20 mmol L^−1^. However, it should be noted that, to prevent NGC contamination, it was tried not to use any further salts to further controlling the ionic strength. In addition, more ionic strength led to have strong influence on the commercial cost and industrial application of the NGC fluids. Fortunately, at this condition, acceptable chloride removal efficiency was evaluated for the NGC samples.

### Effect of Temperature on the Extraction Process

Clearly, besides the thermodynamic equilibrium constant, the kinetics of the reactions strongly depends on the temperature of the reactions. However, in a flammable and volatile sample such as NGC, high temperature was not so safe for the laboratory experiments. Therefore, for more safety and for more simplicity, it was recommended to operate the removal process at room temperature. Providentially, the chloride removal efficiency in this study was so high that small fluctuation in the temperature seemed to have no significant effect(s) on the chloride removal efficiency of the NGC samples.

### Figures of Merit

The CV-AAS method was utilized to determine the concentration of Hg(0) the same as 231 ± 15 ng ml^−1^ (*n* = 3) in the raw NGC. This result pointed to the serious effect(s) of Hg(0) in the condensate. Hence, after treating the NGC with the nano-emulsion at the optimum condition, extraction (mass transfer) of the Hg(0) from the NGC phase to the aqueous one was followed during applying the de-emulsification process. At this condition, change in the oxidation number of the mercuric species from zero to the Hg_2_^2+^ and/or Hg^2+^ using oxidants like (for instance H_2_O_2_) could seriously limit the toxicity and danger of mercury inside the receiving aqueous solution.

The IEC analysis also revealed the Hg^2+^ concentration in the aqueous phase to be 228 ± 6 ng ml^−1^ (*n* = 3). The result was in good agreement with the Hg^2+^ concentration, estimated using the CV-AAS (218 ± 7 ng ml^−1^, *n* = 3). However, for more confidence, the CV-AAS analytical process on the treated NGC revealed the residual Hg concentration to be below 10.0 ng ml^−1^.

For the uncertainty of the Hg content in the NGC, this value is related to the reproducibility of the mercuric element. For the efficiency, we deal with repeatability (for single NGC sample). In addition, for this analysis, we handle the CV-AAS, as a reliable method with detection limit less than ∼2 ng ml^−1^ and precision (reproducibility, RSD %) below around 4.0%. In addition, the statistical test such as (*t*-test) points to the presence or absence of any the Hg levels. Hence, the selected method for mercury removal showed removal efficiency between 90% and around 100% (deepening on the nature of the NGC). This pointed to its effective capability for removal of toxic reagents such as Hg(0) from the NGC samples along with their transfer and protected into a viscose fluid like oil medium.

Nevertheless, to confirm this claim, the IEC analyses also pointed to small concentration of the ${\text{Cl}}^{-}$ inside the treated NGC (below 20.0 ng ml^−1^), compared to the raw ones (315 ± 4) μg ml^−1^) that again exhibited >90% ${\text{Cl}}^{-}$ removal efficiency (see ion-exchange chromatogram and image analyses, Supplementary Figure S2). On the basis of the maximum time interval of the ${\text{Cl}}^{-}$ and Hg(0) removal from the NGC, the extraction time was estimated below 3.0 min. However, this value was dependent to the volume ratio of the gas condensate and the nano-emulsion phase. Consequently, the recommended method in this study again showed effective capability to remove both ${\text{Cl}}^{-}$ and Hg(0) at standard temperature and pressure (i.e., 0°C and 1.0 atm. pressure).

#### Reusability of the Synthesized Nano-Emulsion

The reusability of the synthesized nano-emulsion was also evaluated. The experiments showed that the synthesized nano-emulsion at the optimum ${\text{Cu}}^{2+}$ and oil concentrations could be reused for at least four sequential analyses during extracting the ${\text{Cl}}^{-}$ ions from the NGC, sequentially. After that the efficiency of extraction would practically decrease, as it could not be reused for long time, owing to partial increase in the ${\text{Cu}}^{2+}$ concentration in the treated NGC. However, this can be almost compensated *via* R_22_ purging based on the recommended procedure. Subsequently, the ${\text{Cl}}^{-}$ and Hg(0) removal process can be managed in a returned removal cycle, without any major need(s) to adopt extra nano-emulsion as the extracting phase (see Supporting Information, Supplementary Material S2.3).

#### Selectivity of the Extraction Process

**FIGURE 2:**
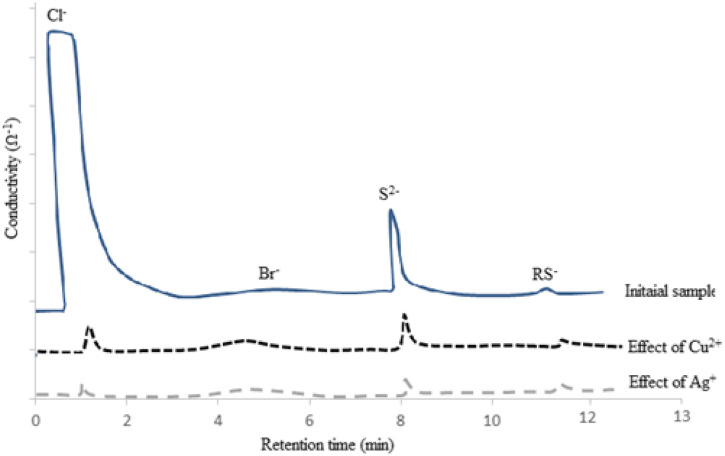
Ion-exchange chromatograms showing selectivity as well as effects of Cu^2+^ and Ag^+^ on Cl^−^ and S^2−^ removal.

The selectivity of this method was evaluated during extraction and separation of different kinds of anions such as Cl^−^, Br^−^, and S^2−^ and trace quantity of RS^−^, presented in the NGC matrix according to chronograms shown in Figure 2.

On the basis of the IECs, not only the capability of the method was investigated for chloride removal purposes but also the effective role of de-emulsifying agents such as Cu^2+^ and Ag^+^ has been studied. On the basis of the results, the synthesized nano-emulsion had enough capability for removal of anionic species such as Cl^−^ and S^2−^. For the Br^−^ and RS^−^ ions, probably large sizes and trace quantity of these anions limited their removal by using this LLE technique.

#### Non-corrosive Property of the NGC

**FIGURE 3:**
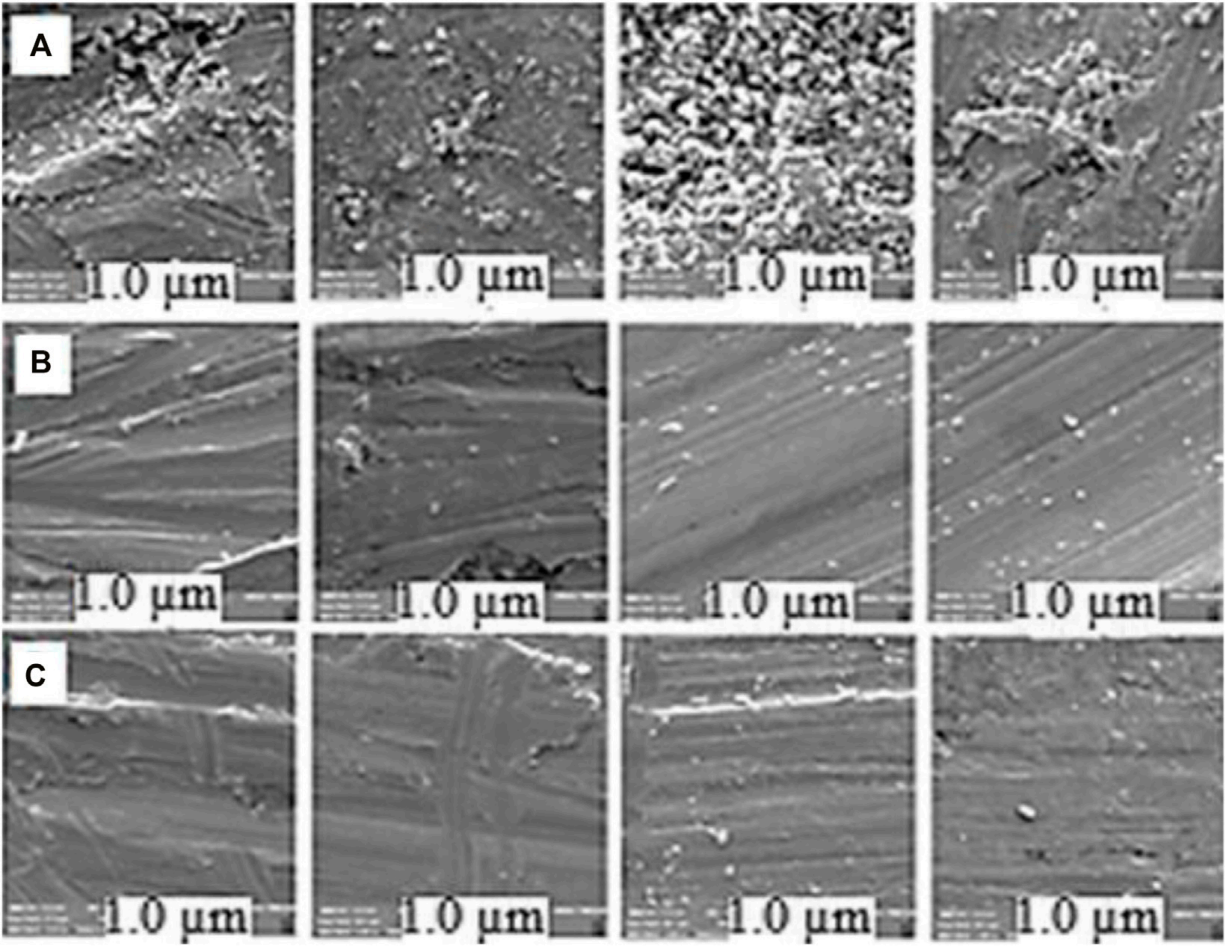
Row FE-SEM images of iron fragments, placed inside row **(A)** pristine NGC and row **(B)** treated condensate. Row **(C)** FE-SEM images of fresh iron fragments as blanking control.

Corrosion test was performed to investigate the ${\text{Cl}}^{-}$ and Hg(0) removal efficiency of the treated NGC. For this purpose, weight loss analysis and direct visualization by field-emission SEM (FE-SEM) of some iron fragments, under similar conditions. This process was achieved *via* situation inside both treated (Figure 3, row A) and pristine (raw) NGC (Figure 3, row B). The results were compared to the untreated one as the blanking control (Figure 3, row C).

As shown (Figure 3), no significant change was observed on the surface structure of the treated Fe fragments versus the untreated and control ones. In addition, no major weight loss was observed in the Fe fragments, contacted to the treated NGC, opposed to the control ones, which were introduced to the pristine NGC under similar conditions.

According to the weight loss analysis, the non-corrosion efficiency was also defined as the mass gradient (weight loss) of the Fe fragment before and after contact with the NGC at a fixed time interval vs. the mass of the fresh fragment, × 100. In this experiment, this value was estimated to be >90%. Therefore, the performed extraction methodology on the pristine NGC was successful for simultaneous removing of both ${\text{Cl}}^{-}$ and Hg(0) and majorly promotion of the non-corrosion efficiency.

### Proposed Mechanisms

**FIGURE 4:**
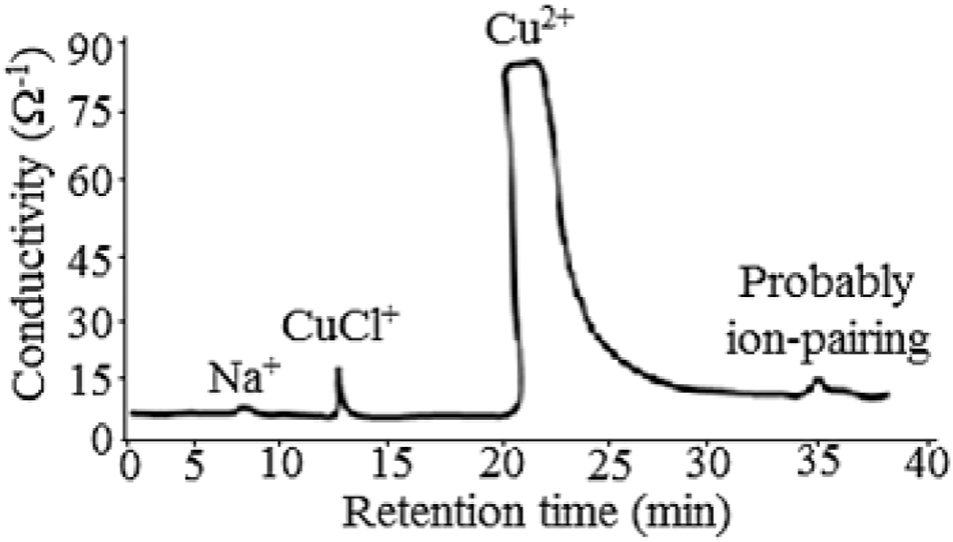
Chromatogram showing the probable mechanism of the method for the ${\text{Cl}}^{-}$ removal process.

Because of the sophisticated matrix of the condensate, it was not easy to report certain mechanism for the Hg(0) and the ${\text{Cl}}^{-}$ removal process in this method. Nevertheless, a probable mechanism has been proposed according to some experimental and theoretical evidence. This hypothetical proposal was almost based on focusing on the formation constants of copper and chloride ions (Zhang et al., 2014). However, on the basis of the results evaluated in the previous sections, it seemed that the synthesized nano-emulsion played role as an intermediate for effective interaction between Cu^2+^ in aqueous phase and Cl^−^ in the NGC phase. This behavior could be related to the interaction among water, sweet oil, and R_22_. To interpret the intermediate species, the effects of formation of different copper species such as Cu^2+^, CuCl^+^, CuCl_2_, CuCl_3_^−^, and CuCl_4_^2−^ were evaluated. These estimations were based on different analytical calculations such as cation-exchange chromatography (IEC, Figure 4).

In Figure 4, we adopted the IEC system that utilized suppressor. This module played role as multiple (at least four) ports: *1*) memory effect elimination, *2*) pH controlling system, *3*) ionic strength controlling, and *4*) a transient value for the transport of the samples toward the EC detector. These features were automatically arranged *via* controlling the mass transfer of different liquid media such as buffer solution and solvent. In this system, for the retention time of 20.0 min, the sensitivity of the system for the Cl^−^ removal (at large scale) was adjusted *via* setting the mass transfer broadening of the analyte using the suppressor. This was achieved to rapidly control the mass transfer of the analyte at the interface (dead volume) between the column and the EC detector. This caused to more reliably analyze the responsible species as maximum as possible. That is why, in Figure 4, as clearly seen, the onset peak at 20 min was tilted backward toward the *Y* axis. This process was consequently general in the chromatographic systems. On the basis of this result, the synthesized nano-emulsion was considered as suitable intermediate medium for effective interaction between the ${\text{Cu}}^{2+}$ (in the nano-emulsion phase) and the ${\text{Cl}}^{-}$ (in the condensate phase). This probably caused the formation of the ${\text{CuCl}}^{+}$ as the intermediate species.

For this purpose, the molar concentrations of the species were estimated at different chloride concentrations based on the mole fractions and their thermodynamic constants at the aqueous solution at room temperature. This process was provided using a program the written Visual Basic 6. This program was based on the related mass/charge balance equations (Zhang et al., 2014) (see Embedded Visual Basic 6, VB_6_ program, Zip Files). Equations of formation copper(II) chloride complexes and their formation constants are shown in Eqs. 1–(4 (Zhang et al., 2014). In addition, other prediction and some different theoretical calculations for the estimated mole fractions of each probably generated species majorly promoted this proposed mechanism.

$${\text{Cu}}^{2+}+{\text{Cl}}^{-}⇄{\text{CuCl}}^{+}{\text{ k}}_{\text{f}}=1.86$$ (1)

$${\text{CuCl}}^{+}+{\text{Cl}}^{-}⇄{\text{CuCl}}_{2}{\text{ k}}_{\text{f}}=2.34\times 10^{-1}$$ (2)

$${\text{CuCl}}_{2}+{\text{Cl}}^{-}⇄{\text{CuCl}}_{3}^{-}{\text{ k}}_{\text{f}}=3.63\times 10^{-3}$$ (3)

$${\text{CuCl}}_{3}^{-}+{\text{Cl}}^{-}⇄{\text{CuCl}}_{4}^{2-}{\text{k}}_{\text{f}}=1.26\times 10^{-6}$$ (4)

On the basis of the results, molar ratio of species such as CuCl_2_, CuCl_3_^−^, and CuCl_4_^2−^ was very low (order of below 10^−15^ mol L^−1^). Whereas molar concentrations of species such as Cu^2+^ and CuCl^+^ had been estimated at the order of about 10^−4^ mol L^−1^. This result was probably in good agreement with the probable formation of CuCl^+^, estimated by the IEC as shown in Figure 4.

However, this result also pointed to the effective role of the ${\text{Cu}}^{2+}$ for interaction with the ${\text{Cl}}^{-}$ in the raw NGC. Whereas, in the Hg(0) removal process, the lack of adopting any oxidant(s) for oxidation of Hg(0) to Hg(II) in this method effectively revealed the probability of the presence of electrochemical interaction(s) and complex formation between Hg and ${\text{Cl}}^{-}$ as cathodic and anodic half reactions, respectively (see Supporting Information, Supplementary Equations S1–S6). These were in agreement with other reports (Milazzo et al., 1978; Yan, 1987).

Nevertheless, precisely talking about the mechanism of extraction needs confidence knowledge about other factors such as role of R_22_, behavior of -NH_2_ functional group in oil and/or formation of other intermediates like ion-pairing. These were considered as a good topic for future studies in this field. The proposed mechanism for the effect of the -NH_2_ functional group is shown in Eq. 5. In addition, the proposed mechanism for the effect of ion-pairing formation is shown in Eq. 6, where M^n+^ is ion pair of Cl^−^ in the NGC.

$${\text{Cu}}^{2+}+{\text{Cl}}^{-}+{\text{NH}}_{2}⇄{\text{Cu}}_{2}{\left(\text{OH}\right)}_{3}\text{Cl}$$ (5)

$${\text{Cu}}^{2+}+{\text{Cl}}^{-}+{\text{M}}^{\text{n}+}⇄{\text{Cu}}_{\text{X}}{\left(\text{M}\right)}_{\text{Y}}{\text{Cl}}_{\text{Z}}$$ (6)

### Formation of Dicopper Chloride Trihydroxide Nanoparticles

Another part of this study was related to the formation of the Cu_2_(OH)_3_Cl nanoparticles by the precipitation process after addition of NH_3_ solution (25%) for controlling the acidity value to ∼7.5. The formation of these nanoparticles was achieved after applying the extraction process on the NGC samples according to the recommended procedure. On the basis of Eq. 4.7 (Tanimizu et al., 2007), removed chloride ion in the presence of Cu^2+^ and OH^−^ led to synthesize Cu_2_(OH)_3_Cl particles based on the following reaction (Eq. 7):

$${2\text{Cu}}^{2+}+{\text{Cl}}^{-}+3{\text{OH}}^{-}⇄{\text{Cu}}_{2}{\left(\text{OH}\right)}_{3}{\text{Cl}}_{\left(\text{s}\right)}$$ (7)

Effective factors such as Cu^2+^ concentration and the acidity were considered as the most important factor during the synthesis of Cu_2_(OH)_3_Cl nanoparticles.

#### Effect of Acidity

**FIGURE 5:**
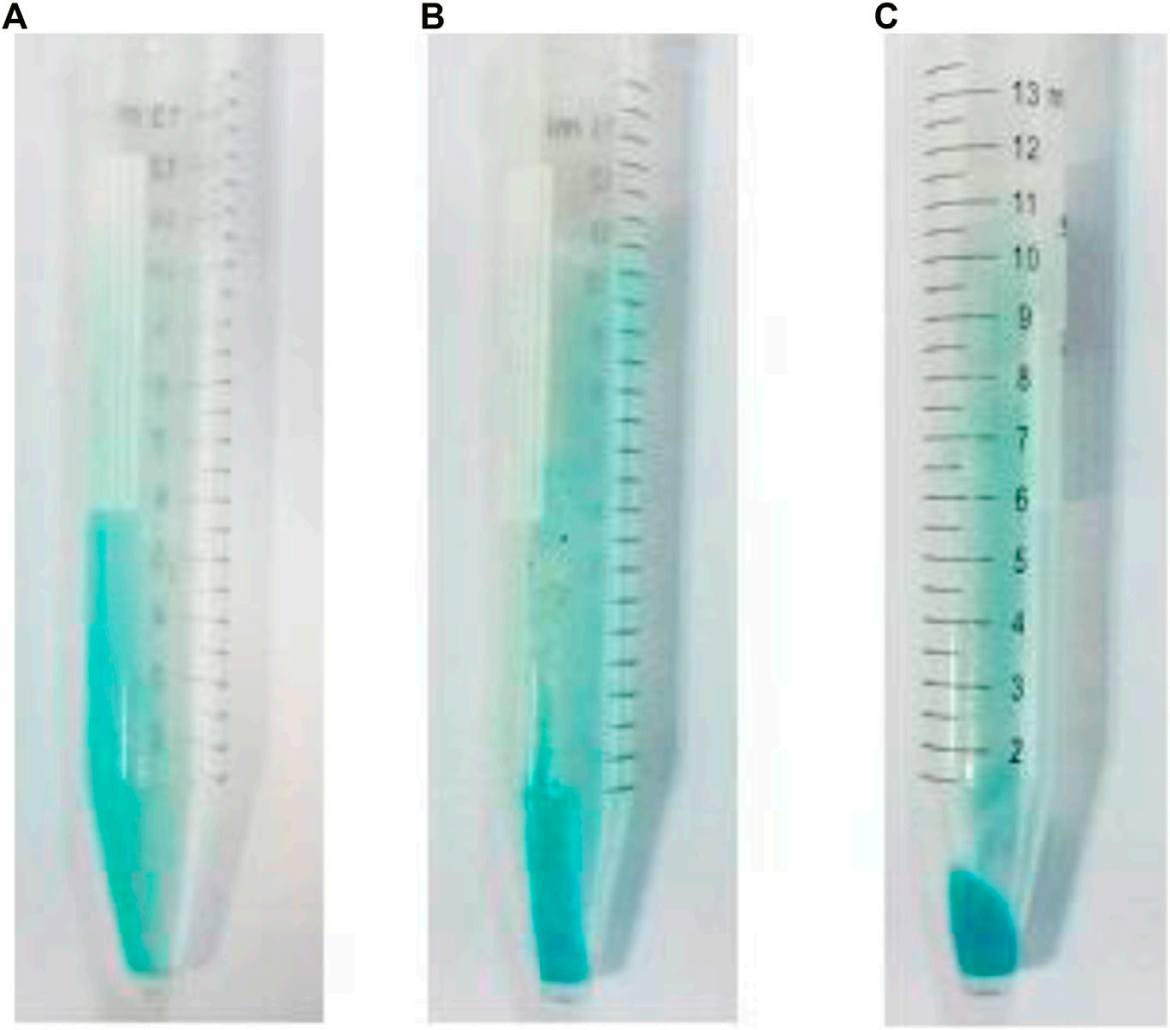
Effect of pH on workup. The pH condition: **(A)** 7 , **(B)** 8, and **(C)** 9.

At pH values higher than ∼7.5, Cu_2_(OH)_3_Cl was unstable and could be converted to Cu(OH)_2_ (Eq. 8) (Frost et al., 2002; Boita et al., 2014). Therefore, pH controlling was very important factor in the workup step. Figure 5 shows the precipitate in different pH values.

Consequently, a pH value as large as ∼7.5 (that was adjusted by commercial NH_3_ solution, purity 25.0%, W/W) was selected. On the basis of the images, the promotion of pH from ∼7.5 to ∼9.0 led to have dark color precipitation due to the formation of Cu(OH)_2_ according to Eq. 8:

$${\text{Cu}}_{2}{\left(\text{OH}\right)}_{3}{\text{Cl}}_{\left(\text{s}\right)}+{\text{OH}}^{-}⇄2\text{Cu}{\left(\text{OH}\right)}_{2\left(\text{s}\right)}+{\text{Cl}}^{-}$$ (8)

#### Effect of Different Bases on the Sedimentation Process

The effects of different bases such as NaOH and NH_3_ were studied. Sedimentation was occurred in both cases, but NH_3_ was adopted for pH controlling in the workup step for facilitation of the procedure. This was due to the effectiveness of NH_3_ for buffering the solution. However, the buffer capacity of NH_3_ was low at pH ∼7.5, but some side effects like formation of by-product precipitation of other buffer compounds such as phosphate limited the use of other buffer species.

#### Effect of Cu^2+^ as de-emulsifying Reagent on the Formation of Dicopper Chloride Trihydroxide Nanoparticles

**FIGURE 6:**
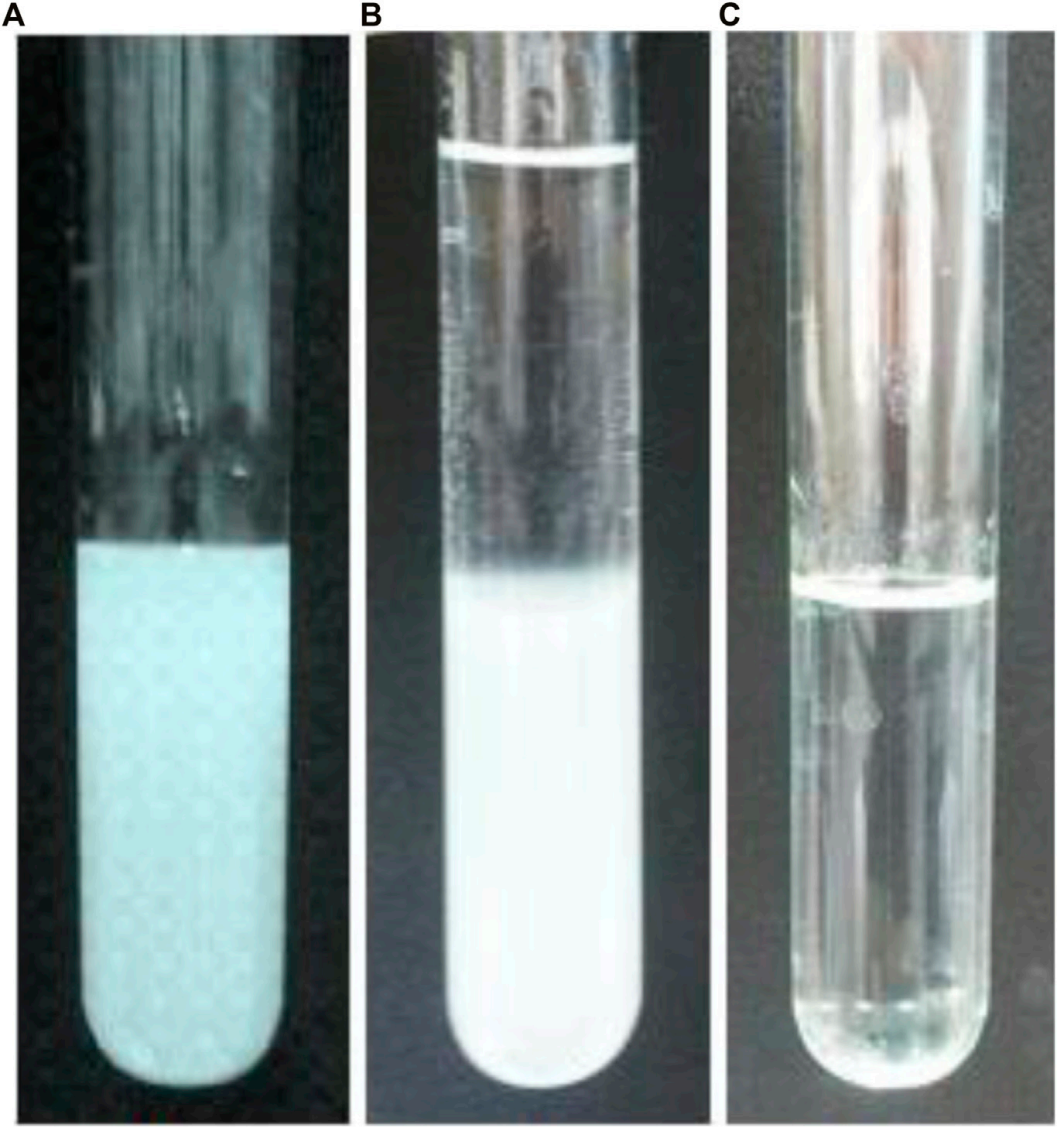
Photographic images showing **(A)** nano-emulsion (oil, 6.6 × 10^−3^ mol L^−1^ Cu^2+^ and 10,400.0 μg ml^−1^) before extraction, **(B)** nano-emulsion after extraction (before demulsifying step), and **(C)** solution after applying the de-emulsification process.

To form the precipitation, after extraction step by nano-emulsion, the extractant medium (nano-emulsion) was separated and Cu(NO_3_)_2_ salt was added to it to increase the Cu^2+^ concentration up to about 1.7 × 10^−2^ mol L^−1^. Then, it was centrifuged for 5 min at 3,000 rpm to demulsify the nano-emulsion and to remove oil from solution. Figure 6A shows the nano-emulsion (oil, 6.6 mmol L^−1^ Cu^2+^ and 10,400.0 μg ml^−1^) before extraction. Whereas, Figure 6B shows the nano-emulsion after extraction (before demulsifying step). Figure 6C exhibits the solution after applying the de-emulsification process on the NGC samples (i.e., after removing oil from internal wall of sample tube). This was therefore ready for addition of NH_3_ and formation the sedimentation.

The addition of NH_3_ should be occurred after applying the de-emulsification during introducing Cu^2+^. At this condition, Cu_2_(OH)_3_Cl nanoparticles were observed in the aqueous phase. On the basis of the direct observation (Figure 6), at least 1.7 × 10^−2^ mol L^−1^ concentration of Cu^2+^ was needed for this purpose during centrifuging for 5 min at 3,000 rpm. This result was in good agreement with Cu^2+^ concentration that was needed for the Cu_2_(OH)_3_Cl formation. It should be noted that increasing Cu^2+^ concentration decreased time and rate of centrifuging step but formed Cu(OH)_2_ and Cu_2_(OH)_3_Cl precipitate. Therefore, these condition was selected as optimum procedure during formation of Cu_2_(OH)_3_Cl nanoparticles.

#### Stability of Dicopper Chloride Trihydroxide Nanoparticles: Effect of Drying Step

Solid-state product was unstable in humidity condition after ∼24 h. This was because this product had probably been converted to Cu(OH)_2_. Therefore, it was recommended to air dry the synthesized sediments by a fan for ∼ 6 h.

#### Characterization of Dicopper Chloride Trihydroxide

In this study, the formation of Cu_2_(OH)_3_Cl was confirmed by the following analytical techniques. Because of the presence of competition between the formation of Cu_2_(OH)_3_Cl and Cu(OH)_2_, sometimes, characters of Cu_2_(OH)_3_Cl were needed to be compared to the Cu(OH)_2_. On the basis of the literature, there are several reactions in which Cu_2_(OH)_3_Cl and Cu(OH)_2_ can compete with each other. Some of the reactions are shown in Eqs 9–12 (Frost et al., 2002; Boita et al., 2014):

$${\text{Cu}}_{2}{\left(\text{OH}\right)}_{3}\text{Cl}+3\text{NaOH}⇄2\text{Cu}{\left(\text{OH}\right)}_{2}+\text{NaCl}$$ (9)

$${\text{Cu}}_{2}{\left(\text{OH}\right)}_{3}\text{Cl}+3\text{HCl}⇄2{\text{CuCl}}_{2}+3{\text{H}}_{2}\text{O}$$ (10)

$${2\text{CuCl}}_{2}+3\text{NaOH}⇄{\text{Cu}}_{2}{\left(\text{OH}\right)}_{3}\text{Cl}+3\text{NaCl}$$ (11)

$${\text{CuCl}}_{2}+3{\text{CuO}+3\text{H}}_{2}\text{O}⇄2{\text{Cu}}_{2}{\left(\text{OH}\right)}_{3}\text{Cl}$$ (12)

According these equations, the structures of Cu(OH)_2_ and Cu_2_(OH)_3_Cl should be comprised in detail.

#### Comparison Between the Structures of Dicopper Chloride Trihydroxide and Copper Hydroxide

**FIGURE 7:**
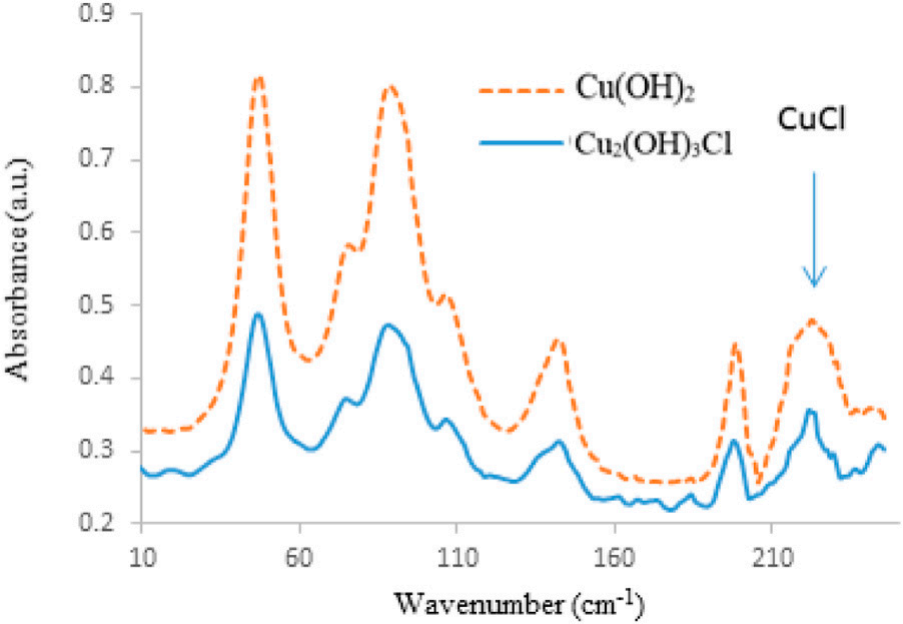
Far FT-IR spectra of Cu_2_(OH)_3_Cl and Cu(OH)_2_.

For precise evaluation of the difference between Cu_2_(OH)_3_Cl and Cu(OH)_2_, the structures of these two compounds were compared to each other (Frost et al., 2002; Boita et al., 2014). On the basis of these references, the Cu_2_(OH)_3_Cl compound consisted of functional groups such as Cu-O, O-H, and Cu-Cl. Cu^2+^ had been coordinated with -OH and -Cl. In addition, the O-H bonds have been bridged through the hydrogen bonding; whereas, for the Cu(OH)_2_, there were some functional groups such as Cu-O and O-H bonds. Consequently, compared to the Cu_2_(OH)_3_Cl, the difference between the functional groups was only related to the presence of Cu-Cl functional group in the Cu_2_(OH)_3_Cl compound. Far Fourier transform infrared (FT-IR) spectra of Cu_2_(OH)_3_Cl and Cu(OH)_2_ are shown in Figure 7.

**FIGURE 8:**
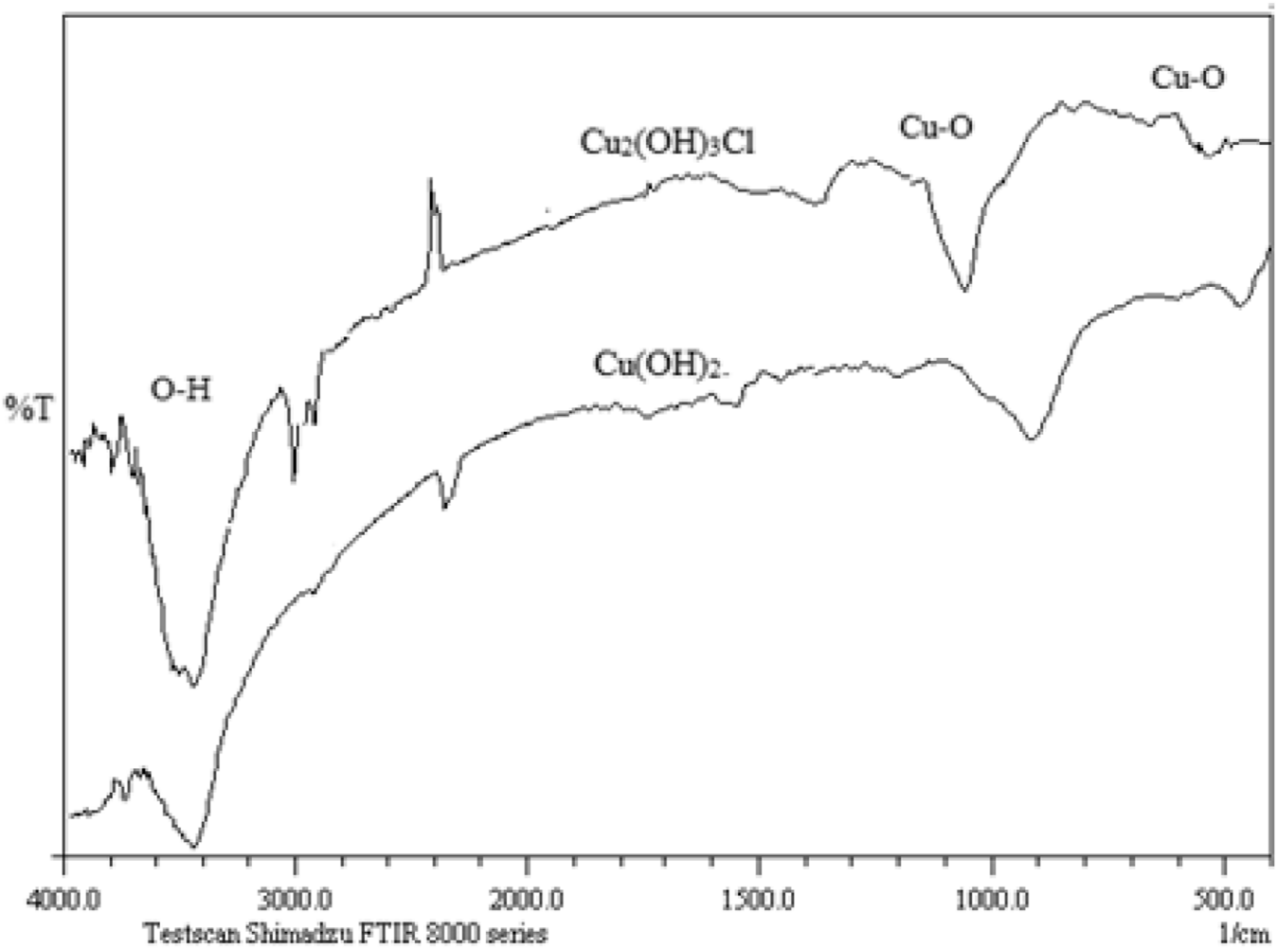
Middle FT-IR spectra of Cu_2_(OH)_3_Cl and Cu(OH)_2_.

No significant difference was observed during compassion between the Far FT-IR spectra of Cu_2_(OH)_3_Cl and Cu(OH)_2_. However, small difference was exhibited between the Cu-Cl functional groups at frequencies of 245–250 cm^−1^. The presence of several shoulders at this frequency range pointed to the presence of different Cu-Cl bonds in the synthesized nanoparticles vs. other copper species such as Cu(OH)_2_. However, for characterization of the sample with more detail, middle FT-IR spectrometry was utilized (Figure 8).

On the basis of the middle FT-IR spectra, the observation of strong peaks at frequencies of 473 and ∼825 cm^−1^ was related to the Cu-O functional group of Cu(OH)_2_; whereas the observation of significant shift to higher frequencies was probably related to the presence of hydrogen bond in the Cu_2_(OH)_3_Cl compound. Compared to the Cu(OH)_2_, the presence of two split absorption bonds at frequency of around 3,420 cm^−1^ clearly pointed to the presence of hydrogen bonds in Cu_2_(OH)_3_Cl.

**FIGURE 9:**
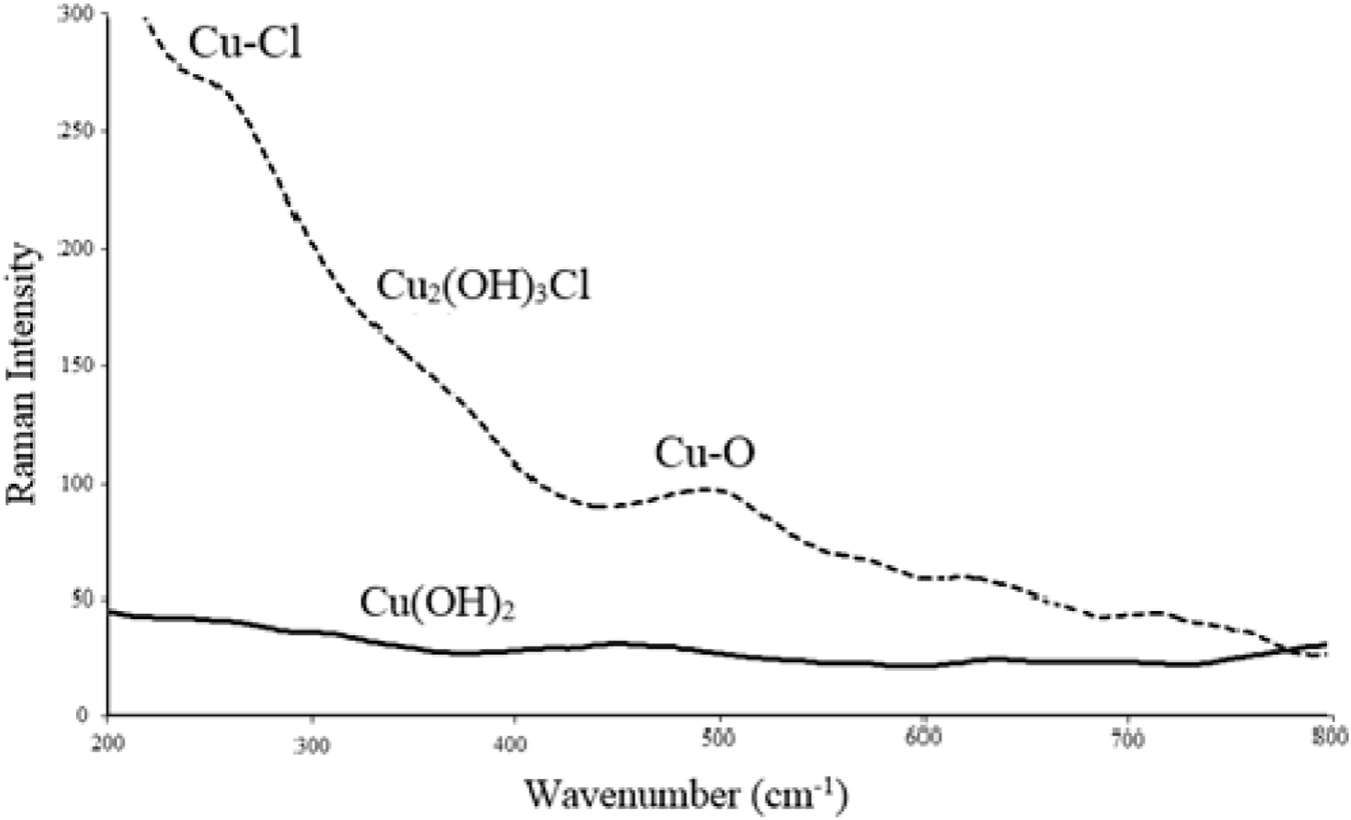
Raman spectra of Cu_2_(OH)_3_Cl and Cu(OH)_2_.

Raman spectroscopy was also considered as another applicable tool for characterization of Cu_2_(OH)_3_Cl, as shown in Figure 9 (Thermo Nicolet FT-Raman, 670). The presence of significant peak at frequency of around 250 cm^−1^ was attributed to the Cu-Cl functional group, which was in good agreement with that estimated in literature (Frost et al., 2002; Boita et al., 2014). Compared to Cu(OH)_2_, major shift was observed on the Raman spectra at frequencies between 450 and 500 cm^−1^. This result again differentiated the formation of Cu_2_(OH)_3_Cl from Cu(OH)_2_.

**FIGURE 10:**
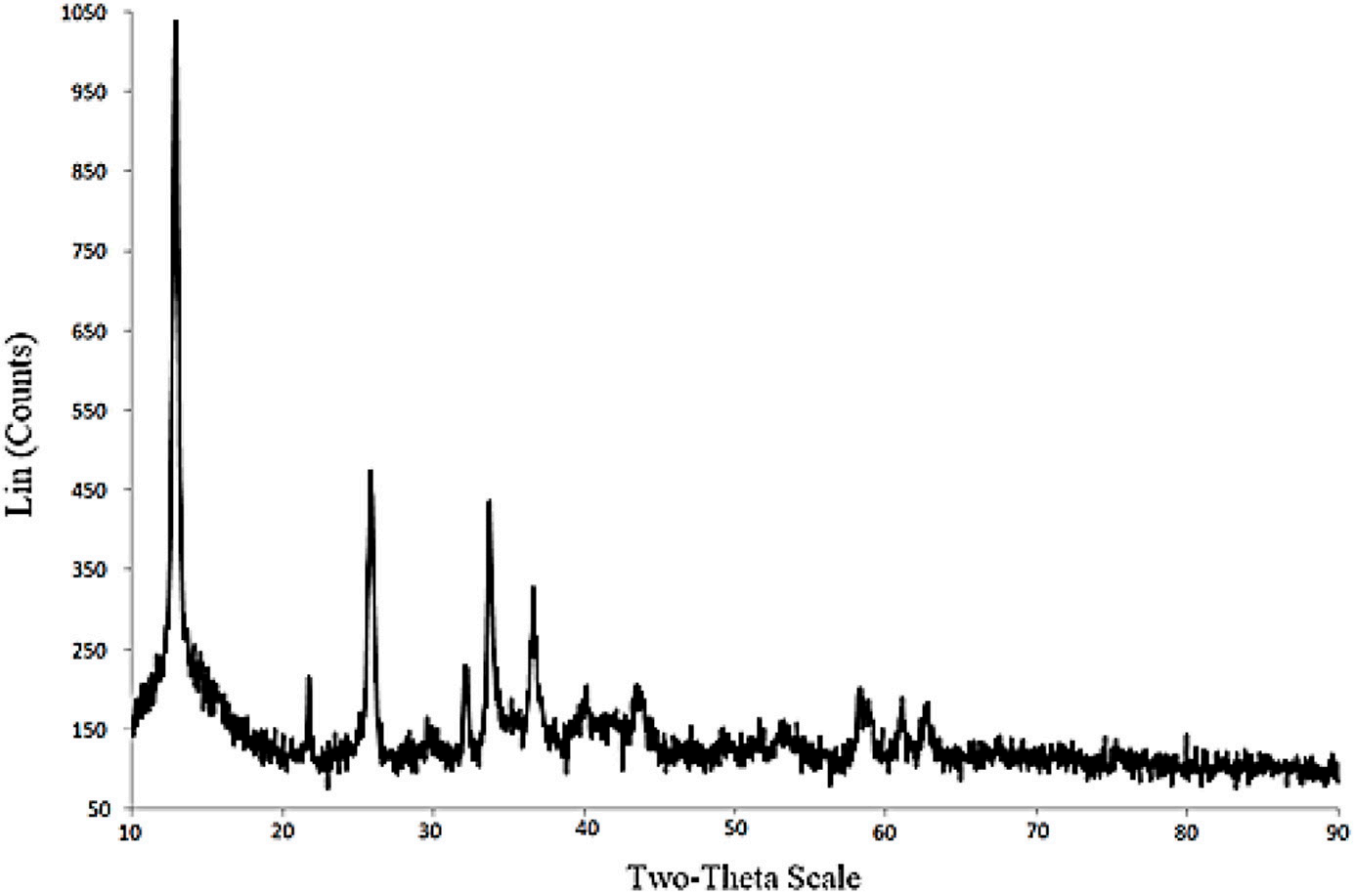
XRD pattern of the synthesized Cu_2_(OH)_3_Cl.

The X-ray powder diffraction (XRD; Bruker’s X-ray Diffraction D8-Discover instrument) pattern of the synthesized ${\text{Cu}}_{2}{\left(\text{OH}\right)}_{3}\text{Cl}$ was shown in Figure 10. On the basis of the XRD pattern, the peaks situated at 2θ = 15°, 27°, 32°, 34°, 60°, and 64° were, respectively, related to special lattices of the ${\text{Cu}}_{2}{\left(\text{OH}\right)}_{3}\text{Cl}$ structure, which were in accordance with other reports (Boita et al., 2014).

**FIGURE 11:**
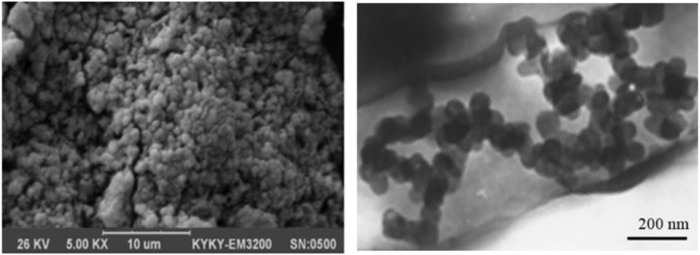
Left: FE-SEM. Right: HR-TEM images of Cu_2_(OH)_3_Cl nanostructures.

The size of Cu_2_(OH)_3_Cl structure was also evaluated; for this purpose, the peak width (W_1/2_) positioned at 2θ = 27° was selected and the crystalline size of the Cu_2_(OH)_3_Cl. This was estimated using Debye–Scherrer equation (Frost et al., 2002). On the basis of this equation, the average size of Cu_2_(OH)_3_Cl was calculated to be ∼150 nm, which is in good agreement with the high-resolution TEM (HR-TEM, Zeiss EM10) and FE-SEM images shown in Figure 11.

**FIGURE 12:**
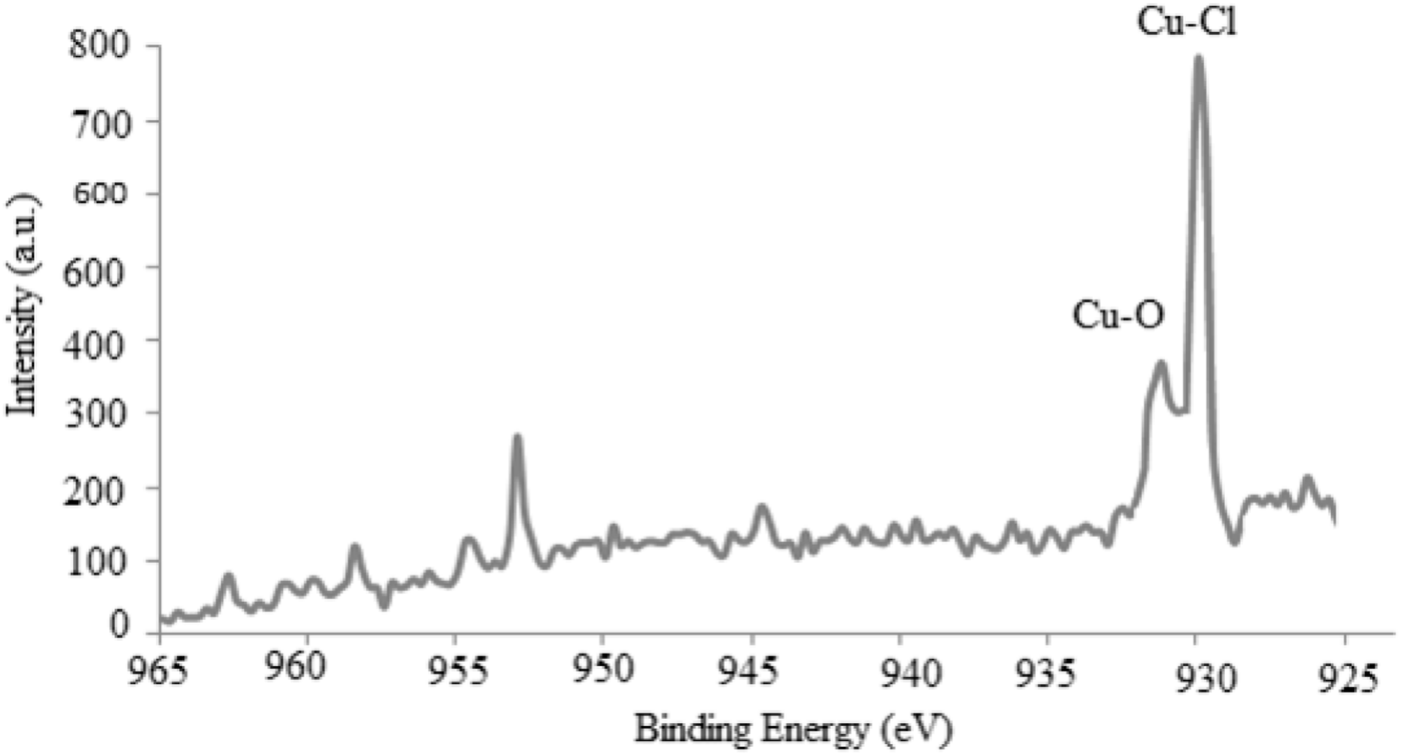
XPS spectrum of the synthesized Cu_2_(OH)_3_Cl nanostructures.

The formation of Cu_2_(OH)_3_Cl was also confirmed using X-ray photoelectron spectroscopy (XPS) analysis (Figure 12). In accordance with the XPS spectrum, the strong peaks, positioned at 952 and 933 eV, were related to the Cu 2P _½_ and Cu 2P_3/2_, respectively, which agreed with those reported in the literature (Boita et al., 2014).

The X-ray fluorescence (XRF) was considered as another important and effective analytical spectroscopy that can be used to estimate the elemental percentages of Cu_2_(OH)_3_Cl. On the basis of the XRF analysis (XRF, Rigaku ZSX Primus II model, rhodium X-ray tube; 4.0-kW maximum power), the weight percentages of the elements presented in the analyzed structure are shown in Table 3. These results pointed to the accuracy of the structure of the analyzed sample during the Cl^−^ removal process.

**TABLE 3:** XRF results with calculation results of weight percentages of the elements in Cu_2_(OH)_3_Cl.

Elements	Elemental percentages (%) by calculation	Elemental percentages (%) by XRF
Cu	59.51	58.74
O	22.47	22.18
Cl	16.60	16.53
C	0.00	0.57
S	0.00	0.24

In addition, CHN-S-Cl elemental analysis was performed to know the molar ratio of elements. The results showed 3.61:2.89:1.03 ratios of H:O:Cl. Therefore, it was hypothesized that precipitation formula was Cu_2_(OH)_3_Cl, not Cu(OH)_2_(H_2_O)_4_ that was predicated with 5.21:3.12 ratios for H:O.

All these characterizations therefore revealed the catalytic property, as well as the nano-template effect of the synthesized nano-emulsion for selective synthesis of the ${\text{Cu}}_{2}{\left(\text{OH}\right)}_{3}\text{Cl}$ nanoparticles with narrow diameter distribution and high purity at room temperature condition.

### Evaluation of the Introduced Method

As explained before, the selectivity of this method was strongly evidenced on the basis of abovementioned documents that revealed high purity of the synthetic product. In addition, the acceptable removal efficiency and the lack of significant memory effect inside the nan-emulsion during its several reusability (at least four times) pointed to the sensitivity of this method. Simple and direct reaction process was also considered as other advantages of this process. Although, the main basis of the synthesized nano-emulsion was the commercial oil; nevertheless, the usability of this extractant strongly reveals the cost-effectiveness of this compound. However, the total price of this compound is not comparable with the economic value of the NGC, especially when resulting in synthesizing a suitable pesticide. Fast reaction time and non-significant toxicity (almost due to the viscose nature of the synthesized nano-emulsion) also made it suitable for such industrial applications.

Fortunately, this synthetic process was not comparable with the previous reports; as (to the best of knowledge) those methods were often suffered from problems such as *1*) high cost, *2*) small efficiency, *3*) long synthetic time, and/or *4*) hard conditions such as high temperature and pressure (Frost et al., 2002; Boita et al., 2014).

## Conclusion

This report introduces an O/W nano-emulsion as the reusable extractant phase using LLE methodology for the removal efficiency of Cl^−^ and Hg(0). The removal efficiency was estimated between 90% and ∼100%, deepening on the nature of the NGC at a brief separation time (<3.0 min). Consequently, serious impurities in condensate were removed by the scalable introduced methodology. In this method, the synthesized nano-emulsion as the extractant phase in the LLE methodology has significant advantages, especially *1*) elemental mercury and chloride ion removal and *2*) selective synthesis of the ${\text{Cu}}_{2}{\left(\text{OH}\right)}_{3}\text{Cl}$ nanoparticles as useful product, specifically as an eco-friendly agricultural pesticide. The achieved safety of the NGC using this nano-emulsion results in efficient reduction in the corrosion rate during testing iron-based fragments (vs. the untreated ones as controls) and increasing of the NGC economic value. All these possibilities are attributed to the intermediate transport properties of the introduced O/W nano-emulsion. At this condition, this reagent plays role as a recycled motor for the NGC purification and conversion of these impurities into the safe and usable products. The introduced method is therefore accepted as direct, simple, low-cost, and scalable conversion of some upstream industries with the downstream ones. To the best of knowledge, this study is considered as the first report for dealing with the chloride and mercury impurities inside the gas condensate.

## Data Availability

The original contributions presented in the study are included in the article/Supplementary Material, further inquiries can be directed to the corresponding author.

## References

[B1] Abai M. Atkins M. P. Hassan A. Holbrey J. D. Kuah Y. Nockemann P. (2015). An Ionic Liquid Process for Mercury Removal from Natural Gas. Dalton Trans. 44, 8617–8624. 10.1039/C4DT03273J 25722100

[B2] Argyle M. Bartholomew C. (2015). Heterogeneous Catalyst Deactivation and Regeneration: A Review. Catalysts 5, 145–269. 10.3390/catal5010145

[B3] Banan Khorshid Z. Mahdi Doroodmand M. Abdollahi S. (2021). UV-vis. Spectrophotometric Method for Oil and Grease Determination in Water, Soil and Different Mediates Based on Emulsion. Microchemical J. 160, 105620. 10.1016/j.microc.2020.105620

[B4] Bingham M. D. (1990). Field Detection and Implications of Mercury in Natural Gas. SPE Prod. Eng. 5, 120–124. 10.2118/19357-pa

[B5] Boita J. do Carmo Martins Alves M. Morais J. (2014). A Reaction Cell for Time-Resolvedin situXAS Studies during Wet Chemical Synthesis: the Cu2(OH)3Cl Case. J. Synchrotron Radiat. 21, 254–258. 10.1107/S1600577513028786 24365945

[B6] Brühl C. A. Bakanov N. Köthe S. Eichler L. Sorg M. Hörren T. (2021). Direct Pesticide Exposure of Insects in Nature Conservation Areas in Germany. Scientific Rep. 11, 24144–24154. 10.1038/s41598-021-03366-w PMC8677746 34916546

[B7] Chen H. Zhong Q. (2022). Physical and Antimicrobial Properties of Self-Emulsified Nanoemulsions Containing Three Synergistic Essential Oils. Int. J. Food Microbiol. 365, 109557. 10.1016/j.ijfoodmicro.2022.109557 35121386

[B8] D'Alfonso C. Lanzalunga O. Lapi A. Vadalà R. (2014). Comparing the Catalytic Efficiency of Ring Substituted 1-hydroxybenzotriazoles as Laccase Mediators. Tetrahedron 70, 3049–3055. 10.1016/j.tet.2014.02.068

[B9] D’Acunzo F. Galli C. (2003). First Evidence of Catalytic Mediation by Phenolic Compounds in the Laccase-Induced Oxidation of Lignin Models. Europ. J. Biochem. 270, 3634–3640. 10.1046/j.1432-1033.2003.03752.x 12919328

[B22] Ergun M. Turan A. Y., Pitting Potential and protection Potential of Carbon Steel for Chloride Ion and the Effectiveness of Different Inhibiting Anions, Corrosion Sci. 32 (1991) 1137–1142. 10.1016/0010-938X(91)90098-A

[B10] FAO/WHO (2021). FAO/WHO Meeting on Pesticide Residues by the 2021 Meeting, 6-17 September; 4 and 7 October 2021.

[B11] Frost R. L. Martens W. N. Rintoul L. Mahmutagic E. Kloprogge J. T. (2002). Raman Spectroscopic Study of Azurite and Malachite at 298 and 77 K. J. Raman Spectrosc. 33, 252–259. 10.1002/jrs.848

[B12] Hong R. Pan T. Qian J. Li H., Synthesis and Surface Modification of ZnO Nanoparticles, Chem. Eng. J. 119 (2006) 71–81. 10.1016/j.cej.2006.03.003

[B13] Hou C. H. Huang C. Y. Hu C. Y. (2013). Application of Capacitive Deionization Technology to the Removal of Sodium Chloride from Aqueous Solutions. Int. J. Environ. Sci. Technol. 10, 753–760. 10.1007/s13762-013-0232-1

[B14] Hussain A. AlAjmi M. F. Rehman M. T. Amir S. Husain F. M. Alsalme A. (2019). Copper(II) Complexes as Potential Anticancer and Nonsteroidal Anti-inflammatory Agents: In Vitro and In Vivo Studies. Sci. Rep. 9, 5237–5254. 10.1038/s41598-019-41063-x 30918270 PMC6437194

[B15] Jackson K. Fulton J. L. (1996). Microemulsions in Supercritical Hydrochlorofluorocarbons. Langmuir 12, 5289–5295. 10.1021/la960210i

[B16] Jiang X. Nešić S. Kinsella B. Brown B. Young D. (2013). Electrochemical Investigation of the Role of Cl−on Localized Carbon Dioxide Corrosion Behavior of Mild Steel. Corrosion 69, 15–24. 10.5006/0620

[B17] Lavela P. Macías C. Ovín Ania M. C. Rasines G. Tirado J. L. Zafra M. C. (2015). Mesoporous Carbon Black-Aerogel Composites with Optimized Properties for the Electro-Assisted Removal of Sodium Chloride from Brackish Water. J. Electroanal. Chem. 50, 42–50. 10.1016/j.jelechem.2015.01.016

[B18] Liu D. Li C. Wu J. Liu Y. (2020). Novel Carbon-Based Sorbents for Elemental Mercury Removal from Gas Streams: A Review. Chem. Eng. J. 391, 123514. 10.1016/j.cej.2019.123514

[B19] Lu L. Wang R. L. Zhang Z. J. Steward F. A. Luo X. Liu B. (2010). Effect of Dietary Supplementation with Copper Sulfate or Tribasic Copper Chloride on the Growth Performance, Liver Copper Concentrations of Broilers Fed in Floor Pens, and Stabilities of Vitamin E and Phytase in Feeds. Biol. Trace Elem. Res. 138, 181–189. 10.1007/s12011-010-8623-3 20174978

[B20] Lubej A. Koloini T. Pohar C. (2004). Industrial Precipitation of Cupric Hydroxy-Salts. Acta Chim. Slov. 51, 751–768.

[B21] Luo X. G. Ji F. Lin Y. X. Steward F. A. Lu L. Liu B. (2005). Effects of Dietary Supplementation with Copper Sulfate or Tribasic Copper Chloride on Broiler Performance, Relative Copper Bioavailability, and Oxidation Stability of Vitamin E in Feed. Poult. Sci. 84, 888–893. 10.1093/ps/84.6.888 15971525

[B23] Milazzo G. Caroli S. Braun R. D. (1978). Tables of Standard Electrode Potentials. J. Electrochem. Soc. 125, 261C. 10.1149/1.2131790

[B24] Mohammadi A. Doroodmand M. M. Sadegh M. M. R. (2016). Selective Speciation of Cr(III) and Cr(VI) by Micro-emulsion Using UV-Vis. Spectrophotometry. J. Textile Sci. Eng. 6, 1–6. 10.4172/2165-8064.1000243

[B25] Pathania R. Najda A. Chawla P. Kaushik R. Khan M. A. (2022). Low-energy Assisted Sodium Alginate Stabilized Phyllanthus Niruri Extract Nanoemulsion: Characterization, *In Vitro* Antioxidant and Antimicrobial Application. Biotechnol. Rep. 33, e00711. 10.1016/j.btre.2022.e00711 PMC8850680 35198420

[B26] Pierce R. A. Campbell-Kelly R. P. Visser A. E. Laurinat J. E. (2007). Removal of Chloride from Acidic Solutions Using NO2. Ind. Eng. Chem. Res. 46, 2372–2376. 10.1021/ie061167p

[B27] Sabbaghi S. Maleki R. Shariaty-Niassar M. Zerafat M. M. Nematollahi M. M. (2012). Modeling of Chloride Ion Separation by Nanofiltration Using Machine Learning Techniques. Int. J. Nanosci. Nanotechnol. 8, 185–190.

[B28] Scott D. A. (2000). A Review of Copper Chlorides and Related Salts in Bronze Corrosion and as Painting Pigments. Stud. Conservation 45, 39–53. 10.1179/sic.2000.45.1.39

[B30] Sun W.-Q. Xu X.-D. Zhang Y. Wu J.-Z. (2019). Chlorine Corrosion of Blast Furnace Gas Pipelines: Analysis from thermal Perspective. J. Min Metall. B Metall. 55, 197–208. 10.2298/JMMB181016028S

[B31] Tanimizu M. Takahashi Y. Nomura M. (2007). Spectroscopic Study on the Anion Exchange Behavior of Cu Chloro-Complexes in HCl Solutions and its Implication to Cu Isotopic Fractionation. Geochem. J. 41, 291–295. 10.2343/geochemj.41.291

[B29] Wilhelm S. M., Risk Analysis for Operation of Aluminum Heat Exchangers Contaminated by Mercury, Proc. Saf. prog. 28 (2009) 259–266. 10.1002/prs.10322

[B32] Wilhelm S. M. Bloom N. (2000). Mercury in Petroleum. Fuel Process. Techn. 63, 1–27. 10.1016/S0378-3820(99)00068-5

[B33] Wu X. Liu Z. Liu X. (2013). Chloride Ion Removal from Zinc Sulfate Aqueous Solution by Electrochemical Method. Hydrometallurgy 134-135, 62–65. 10.1016/j.hydromet.2013.01.017

[B34] Yan T. Y. (1987). Removal of Mercury from Natural Gas and Liquid Hydrocarbons Utilizing Downstream Guard Chabmer.

[B35] Yang H. Xu Z. Fan M. Bland A. E. Judkins R. R. (2007). Adsorbents for Capturing Mercury in Coal-Fired Boiler Flue Gas. J. Hazard. Mater. 146, 1–11. 10.1016/j.jhazmat.2007.04.113 17544578

[B36] Zhang N. Zhou Q. Yin X. Zeng D. (2014). Trace Amounts of Aqueous Copper(II) Chloride Complexes in Hypersaline Solutions: Spectrophotometric and Thermodynamic Studies. J. Solution Chem. 43, 326–339. 10.1007/s10953-014-0129-8

