# Supplementary figures and images for: Reusable Extractant and Direct Catalytic Mediation of Water/Oil/Chlorodifluoromethane Nano-Emulsion in Natural Gas Condensate for Efficient Conversion of Chloride Impurities Into the Dicopper Chloride Trihydroxide Nanoparticles
Source: Front Chem. 2022 Apr 26;10:823357. doi: 10.3389/fchem.2022.823357 (PMC9087797; doi:10.3389/fchem.2022.823357)

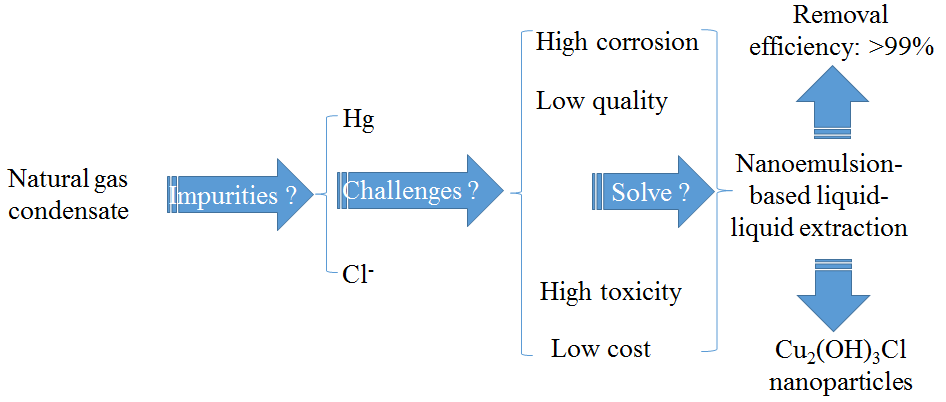

Supplement: Supplementary file 1 [file Image1.TIF]
