# Supplementary material for: Reusable Extractant and Direct Catalytic Mediation of Water/Oil/Chlorodifluoromethane Nano-Emulsion in Natural Gas Condensate for Efficient Conversion of Chloride Impurities Into the Dicopper Chloride Trihydroxide Nanoparticles
Source: Front Chem. 2022 Apr 26;10:823357. doi: 10.3389/fchem.2022.823357 (PMC9087797; doi:10.3389/fchem.2022.823357)
Supplement: Supplementary file 2 [file DataSheet1.docx]

**Reusable extractant and direct catalytic mediation of water/oil/** **chlorodifluoromethane nano-emulsion in natural gas condensate for efficient conversion of chloride impurities into the dicopper chloride trihydroxide nanoparticles**

**Zohre Banan Khorshid, Mohammad Mahdi Doroodmand^*^**

Department of Chemistry, College of Sciences, Shiraz University, Shiraz 71454, Iran

*Corresponding Author

doroodmand@shirazu.ac.ir, [doroodmand@yahoo.com](mailto:doroodmand@yahoo.com), Tel: +098-713-6137152, Fax: +098-713-6460788.

# 1.SI. Introduction

## *1.1.SI. Nano-emulsions properties*

Nano-emulsions have controllable hydrophobic/hydrophilic, well-disciplined emulsification and de-emulsification properties. This compound also possesses special characteristics such as high active surface area and template effect. In addition, design and construction engineering of the nano-emulsion is important to access these efficient characteristics [1]. Recent researches pointed to the strong demand for introduction of novel nano-emulsions with well-behaved characteristics and controllable properties. In this research, therefore different features such as template effect, extracting behavior (as solvent) and catalytic mediation of a novel nano-emulsion are evaluated in detail.

# 2.SI. Experimental

## *2.1.SI.* Reagents and materials

To optimize the kind of cation as the extranet species, different water soluble salts (with analytical grade) including: (CH_3_COO)_2_Mg.4H_2_O, Cu(NO_3_)_2_, Fe(NO_3_)_3_.9H_2_O, Ni(NO_3_)_2_.6H_2_O, Co(NO_3_)_2_.4H_2_O, FeSO_4_.7H_2_O, Mn(NO_3_)_2_.6H_2_O, Ca(NO_3_)_2_.4H_2_O, Al(NO_3_)_3_.9H_2_O, and AgNO_3_ were selected and added to the nano-emulsion, individually.

The raw “*Natural Gas Condensate*” (*NGC*) sample was belong to the Iranian oil and gas fields (Pars Special Energy Economic Zone, Asaluyeh, Bushehr, Iran, coordination: 27°28′34″N 52°36′27″E, Sample date, 2016). Chlorodifluoromethane (CHClF_2_, R_22_) with 99.0 % (W/W) purity percentage was purchased from Australia, Solstice® N40). Light liquid paraffin oil compound with average gram formula weight of 860 ± 10 g mol^-1^, specific gravity of 0.832 (at 25 ^o^C), and dynamic viscosity as large as 27 MPas (at 20 ^o^C), and light absorption intensity the same as <0.08 (a.u.) at 240-280 nm was related to the Eastto, Mumbai Company (India). Triply distilled water was selected as the source of the polar phase during the nano-emulsion synthetic process.

##

## 2.2.SI. Liquid-liquid extraction (LLE)

To operate of the LLE process, 5.0 mL volume of the synthesized nano-emulsion was mixed with the same volume of the NGC sample, and vigorously shook for 1.0 min inside a plastic falcon (Centrifuge Tubes, 13.0 mL, 30 × 115 mm, Screw Cap, Conical, Propylene, No-Leak, Karter Scientific, USA). The falcon was then placed in its holder for 1.0 min to have a short rest; along separating the two immiscible phases, based on their polarity, gravity (i.e. nano-emulsion as the bottom phase) and the NGC as the upper phase.

Subsequently, the NGC was separated and transferred into another falcon using a pipet (10.0 mL) in order to perform both semi-quantitatively (during direct visualization of the generated inorganic particles) and quantitatively (by chromatographic and spectroscopic techniques) tests. Finally, the residual Hg(0) and $\mathrm{Cl}^{-}$ concentrations were estimated in the NGC sample. Further removal processes were also possible after treating the separated nano-emulsion with the R_22_ reagent. Consequently, the $\mathrm{Cl}^{-}$ and Hg(0) removal process can be operated in a returned removal cycle without any major need(s) to add extra amount of nano-emulsion as the extracting phase.

## 2.3.SI*.* Semi-quantitative test for chloride detection

After applying the extraction step, 5.0 mL of the NGC sample was separated and mixed with the same volume of the ${Ag}^{+}$ standard solution (235.0 mmol L^-1^). Then, it was vigorously shaken for 1.0 min inside the plastic falcon. To optimize the concentration of the requisite${Ag}^{+}$, six different concentrations (including 0.352, 0.2350, 0.1175, 0.0587, 0.0294 and 0.0147 mol L^-1^) were individually prepared (5.0 mL). Formation of the AgCl particles was directly visualized, semi-quantitatively, using a charge-coupled device (*CCD*) camera (Samsung S_4_). For better visualization and photographic imaging, the falcon-containing AgCl white particles was positioned on a black background.

## 2.4.SI. Corrosion test

To test the corrosion process, two cubic iron fragments (Type: ca. 323 B.C.–A.D. 256, Western Han Dynasty, China) with 1.0 × 1.0 cm dimension and 0.5 cm width were distinctly introduced to each pristine (raw) and nano-emulsion-treated NGC samples, under similar conditions, along a fourteen-day time interval. Each sample was then analyzed based on the corrosion viewpoint via direct surface observation using scanning electron microscopy (SEM, TESCAN-Vega 3) and weight-loss analysis by a gravimeter (Mettler Toledo™, China).

## 2.5.SI*.* Quantitative tests for mercury determination

Ion chromatography (Ion-exchange chromatography, IEC, ACQUITY UPLC®, Waters, USA) as well as cold vapor-atomic absorption spectroscopy (CV-AAS, Shimadzu, Japan) were adopted to determine the removal percentages of mercuric species from the NGC sample. About the IEC, the aqueous solution (2.0 mL) was diluted 10.0 times using the triply-distilled. The 1.0 mL of the ${Hg}^{2+}$ standard solutions with different concentrations (in the range of 10.0-50.0 mg L^-1^) were individually spiked into the extracted water sample (5.0 mL) based on standard addition method. Then, 0.50 mL of each sample was directly injected to the IEC using a Hamilton syringe (Luer Lock, Trachael, Hamilton Bonaduz^AG^, US). The related peak area in the chromatogram (at a certain retention time) was selected as the detection system.

About the CV-AAS, 20.0 mL of the initial NGC sample was diluted 10.0 times using *n*-pentane (99.0 %, W/W, Merck Company). Then, 0.50 ± 0.01 g ${SnCl}_{2}$ powder (Analytical grade, >99.0 %, W/W, Merck Company) was added and sonicated for 2.0 h with 10.0 MHz frequency using a sonication probe (Fisherbrand™ Q500, UK) to completely reduce any presented the ${Hg}^{2+}$ ions into its elemental form with zero oxidation state (i.e., Hg(0)). Then, the sample was introduced to a continuous/fractional distillation system (H-6173-CDL, "Maleta Cyclic Distillation", LLC OU, Parnumnt 130-38, 11317 Tallinn, Estonia) to separate (evaporate) the Hg(0) vapors from the organic samples. Afterward, 10.0 mL of the gas solution containing Hg vapor was introduced to the CV-AAS system to estimate the quantity of the mercury, presented in the NGC samples.

About the IEC equipment, the columns (20.0 ± 0.1 cm) included ion-exchange resins with –COOH and ${-N(OCH}_{3})H_{3}^{+}$ functional groups for cations and anions, respectively, using a datome-filled column (5.0 cm, Shimadzu, Japan) as guard column. In addition, a fixed mixture of CH_3_OH: H_2_O (high performance liquid chromatography, HPLC, grade 30.0 %, V/V) was selected as the mobile phase at 1100 ± 2 pass pressure and 25 ^o^C temperature. The acidity/basicity (pH) of the mobile phase was controlled at pH 7.0 ± 0.1 using phosphate buffer (0.01 mol L^-1^, Merck Company). The suppressor module was a four–way alternative transmitting clocking pathways including, ionic strength controlling using deionized water, pH buffer condition, mobile phase transition and deionized water as the electrical conductivity (*EC*) detector cleaner.

## 2.6.SI. Formation of $\mathbf{Cu}_{\mathbf{2}}\mathbf{(OH)}_{\mathbf{3}}\mathbf{Cl}_{\mathbf{(s)}}$nanoparticles

De-emulsification step was applied on the nano-emulsion to synthesize the $\mathrm{Cu}_{2}{(OH)}_{3}{Cl}_{(s)}$ nanoparticles. For this purpose, 20.00 ± 0.01 mg $\mathrm{Cu}^{2+}$ (using ${{Cu(NO}_{3})}_{2}$ reagent) was added to the 50.0 mL of the nano-emulsion. Then, the nano-emulsion was introduced to a centrifuge (Andreas Hettich, UK) at 8000 ± 5 rpm for 5.0 min. This procedure resulted in separation of the oil phase from the aqueous phase. This process therefore caused to attach to the inner wall of the test tubes. After that, the basicity (pH) of the aqueous solution was adjusted to 7.5 ± 0.1, slowly, by adding commercial NH_3_ solution (25 %, W/W, Arman-Sina Company), along with following (tracing) the pH values using a litmus paper (litmus paper manufacturers & suppliers, China).

# 3.SI. Results and discussion

## 3.1.SI. Limitations of current analytical methods for the $\mathbf{Cl}^{\mathbf{-}}$and Hg(0) removal

About the NGC, it was impossible to estimate the quantity of the $\mathrm{Cl}^{-}$content using conventional analytical methods such as, for instance, Mohr’s or Fajan’s titrimetric methods, owing to the non-aqueous behavior of the condensate [3]. Also, estimation of the $\mathrm{Cl}^{-}$ concentration in the nano-emulsion phase (after performing extraction) was not feasible using titration methods, even after performing de-emulsification step. This was probably related to the presence of $\mathrm{Cl}^{-}$ ions in their complex forms that resulting in dealing with some gross and significant errors.

Back-titration method was also examined using excess amount of the $\mathrm{Ag}^{+}$ as standard reagent for the analysis of the $\mathrm{Cl}^{-}$ removal from the tested NGC. However, this issue caused significant relative error percentage. This fault was probably related to matrix error and impossibility to fix matrix of the NGC sample during introducing excess amount of the $\mathrm{Ag}^{+}$ as standard medium. About this method, it was necessary to introduce very more excess amount of the $\mathrm{Ag}^{+}$ reagent using standard solution (0.235 mol L^-1^ concentration) that majorly changed the NGC sample matrix. Also, it was impossible to recognize the decreasing in the $\mathrm{Ag}^{+}$ concentration using, for example, a potentiometric titration (by a Ag rod as indicator electrode and Ag/AgCl, ${saturated Cl}^{-}$, as reference electrode), owing to the oxidation/reduction processes problems in the non-aqueous media. Hence, we introduced a simple semi-quantitative test (i.e., directly AgCl particle’s visualization) to recognize the $\mathrm{Cl}^{-}$ existence in the NGC samples during optimizing the related factors by one factor-at-a time method.

###

### 3.2.SI. Optimization of cation and anion for synthesizing nano-emulsion as extractant medium

About the NGC, it was supposed that, presence of a suitable cation in extractant medium (nano-emulsion) could help the extraction of the$\mathrm{Cl}^{-}$ ions from the NGC sample, based on formation insoluble particles. For this purpose, different cations, for example, $\mathrm{Fe}^{3+}$**,** $\mathrm{Mg}^{2+}$**,** $\mathrm{Co}^{2+}$**,** $\mathrm{Al}^{3+}$**,** $\mathrm{Mn}^{2+}$**,** $\mathrm{Fe}^{2+}$**,** $\mathrm{Ca}^{2+}$**,** $\mathrm{Cu}^{2+}$**,** and $\mathrm{Ni}^{2+}$ were contacted with the nano-emulsion-treated NGC sample; their effects on the $\mathrm{Cl}^{-}$ removal were therefore investigated by the semi-quantitative test. Figure S1 (A to I) shows the formation of AgCl particles by adding the $\mathrm{Ag}^{+}$ solution to the treated NGC samples. As clearly shown, only nano-emulsion contained the $\mathrm{Cu}^{2+}$ had good effect on the $\mathrm{Cl}^{-}$ removal from the condensate. This was because of the lack of formation of any further AgCl particle after the $\mathrm{Cu}^{2+}$ introduction.

| **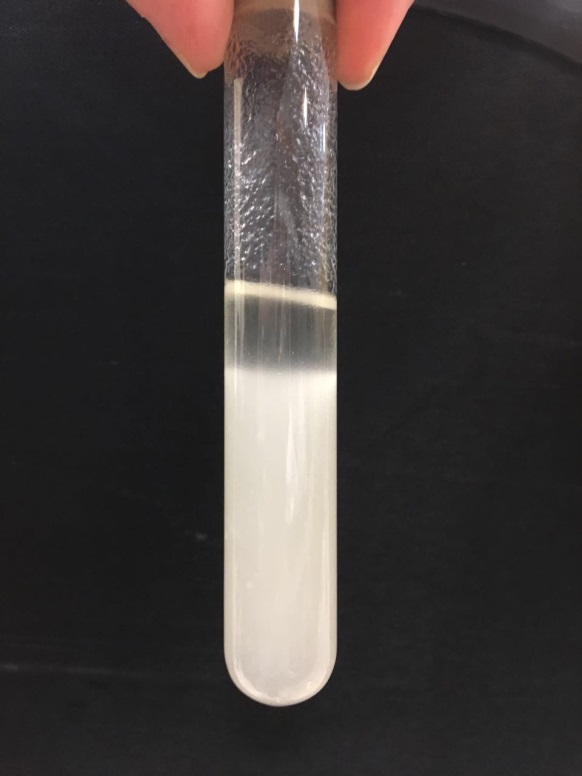** | **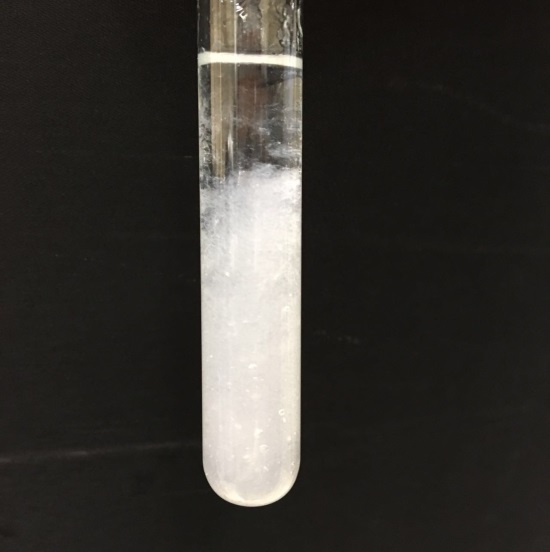** | **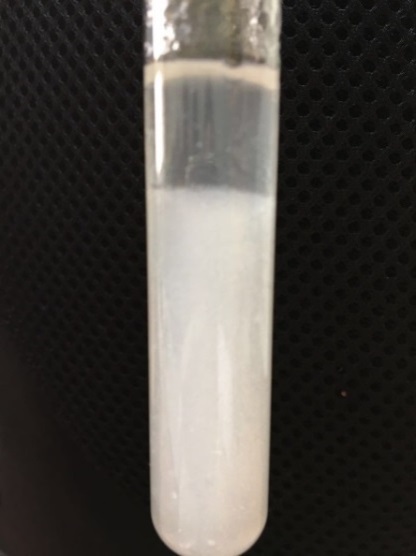** | **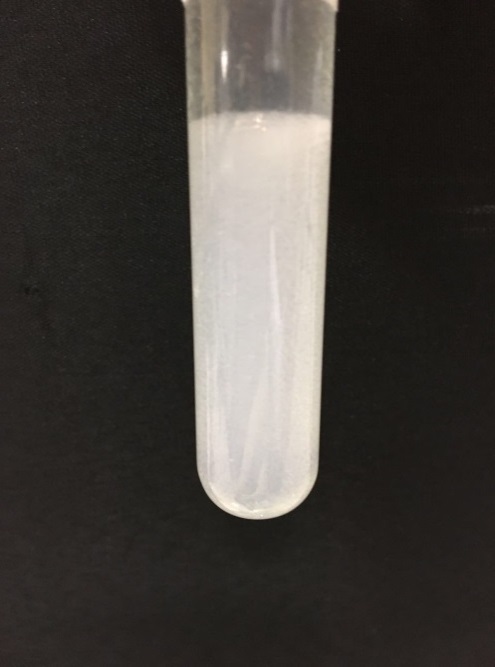** | **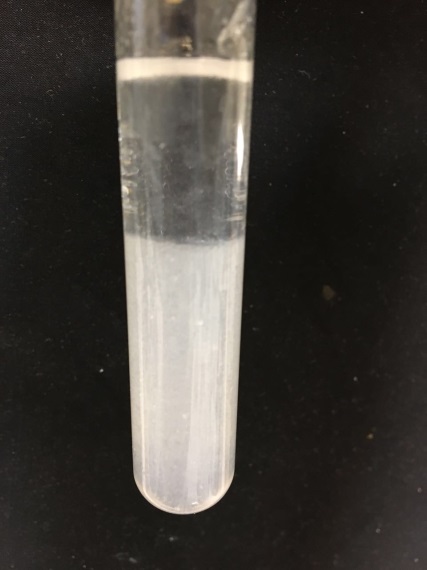** |
| --- | --- | --- | --- | --- |
| **A.** | **B.** | **C.** | **D.** | **E.** |
| **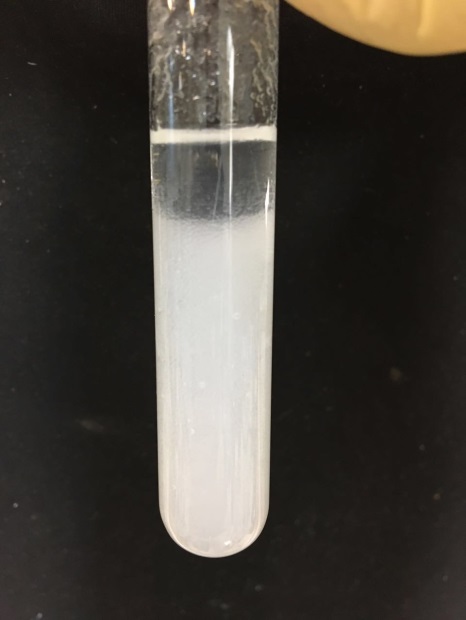** | **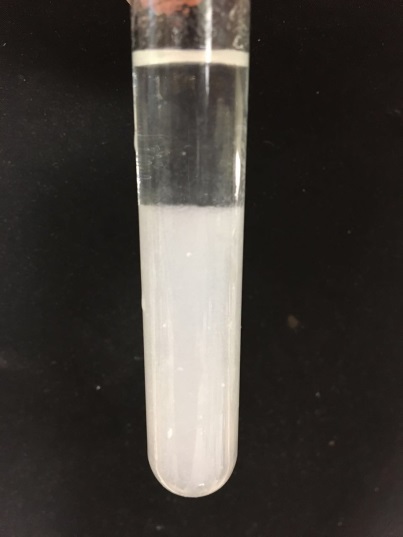** | **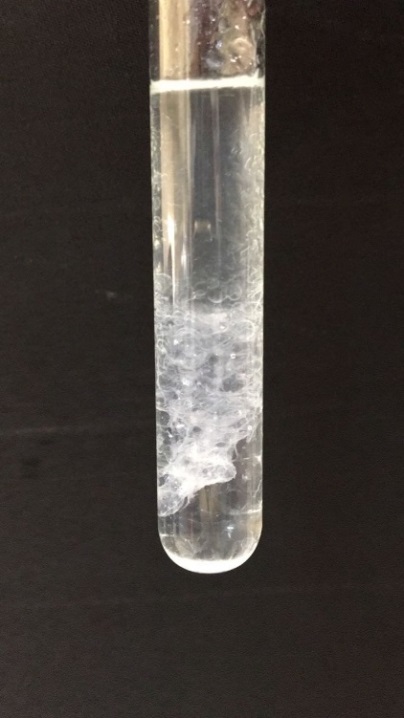** | **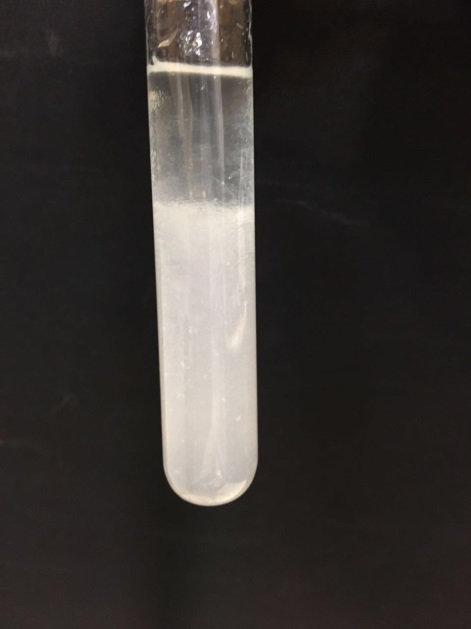** |  |
| **F.** | **G.** | **H.** | **I.** |  |

# Figure S1. | Formation of the AgCl particles after adding the $\mathbf{Ag}^{\mathbf{+}}$ (0.235 mol L^-1^ concentration) to the condensate treated with the nano-emulsion in the presence of different cations: A) $\mathbf{Fe}^{\mathbf{3+}}$, B) $\mathbf{Mg}^{\mathbf{2+}}$, C) $\mathbf{Co}^{\mathbf{2+}}$, D) $\mathbf{Al}^{\mathbf{3+}}$, E) $\mathbf{Mn}^{\mathbf{2+}}$, F) $\mathbf{Fe}^{\mathbf{2+}}$, G) $\mathbf{Ca}^{\mathbf{2+}}$, H) $\mathbf{Cu}^{\mathbf{2+}}$, and I) $\mathbf{Ni}^{\mathbf{2+}}$ (0.02 mol L^-1^ concentration) solutions at room temperature, under similar conditions.

## 3.3.SI. Optimization of the $\mathbf{Ag}^{\mathbf{+}}$ concentration

Various concentrations of the $\mathrm{Ag}^{+}$ solutions (0.3525, 0.2350 and 0.1175 mol L^-1^) had partially the same effect(s) based on the same formation of the AgCl particles in the treated NGC samples, compared to the pristine (raw) NGC sample as control. Besides this, other $\mathrm{Ag}^{+}$ solutions (0.0587, 0.0294, 0.0147 mol L^-1^) were unsuitable, because of low amounts of the generated AgCl particles. So, these tested concentrations were not enough to wholly extract all the presented $\mathrm{Cl}^{-}$ from the NGC sample. Whereas, maximum AgCl particles were observed when introducing $\mathrm{Ag}^{+}$ solution with 0.2350 mol L^-1^ concentration. Therefore, this quantity was selected for the $\mathrm{Ag}^{+}$ solution for fully conversion of $\mathrm{Cl}^{-}$ into the AgCl particles.

## 3.4.SI. Synergistic effect of the $\mathbf{Cu}^{\mathbf{2+}}$ and nano-emulsion

Effect of different cationic species was individually evaluated as both extracting and de-emulsifying agents; as a result of the inappropriate behavior of $\mathrm{Ag}^{+}$ for the extraction of the $\mathrm{Cl}^{-}$ from the nano-emulsion medium to the aqueous phase. Among different cations, $\mathrm{Cu}^{2+}$ was selected, attributable to its excellent synergistic effect with the nano-emulsion, besides its other advantages such as availability and low cost.

In order to evaluate the individual effect of oil and the $\mathrm{Cu}^{2+}$ ions for the $\mathrm{Cl}^{-}$ removal process during the extraction from the NGC samples, three different extractant media were prepared:

-A) Nano-emulsion (10.40 g L^-1^ oil) without any cation,

-B) Aqueous $\mathrm{Cu}^{2+}$ solution (7.4×10^-3^ mol L^-1^),

-And C) Nano-emulsion (10.40 g L^-1^ oil) with the $\mathrm{Cu}^{2+}$ (7.4×10^-3^ mol L^-1^).

Then, during performing of the extraction step, the semi-analytical test was tested, individually. According to Figure S2, neither nano-emulsion (in the absence of any cation(s)) nor the $\mathrm{Cu}^{2+}$ solution (in the nonattendance of any oil medium) had not individually positive effect(s) on the $\mathrm{Cl}^{-}$ removal process. So, these reagents were not independently capable to remove the $\mathrm{Cl}^{-}$ ions, as a result of the presence of the AgCl particles in the NGC sample when applying the semi-quantitative test (Figures S2.A and S2.B). Besides, just nano-emulsion in the presence of the $\mathrm{Cu}^{2+}$ had a worthy effect on the chloride removal process (Figure S2.C). Therefore, nano-emulsion and the $\mathrm{Cu}^{2+}$ had a synergistic effects for the $\mathrm{Cl}^{-}$ removal from the NGC medium.

| 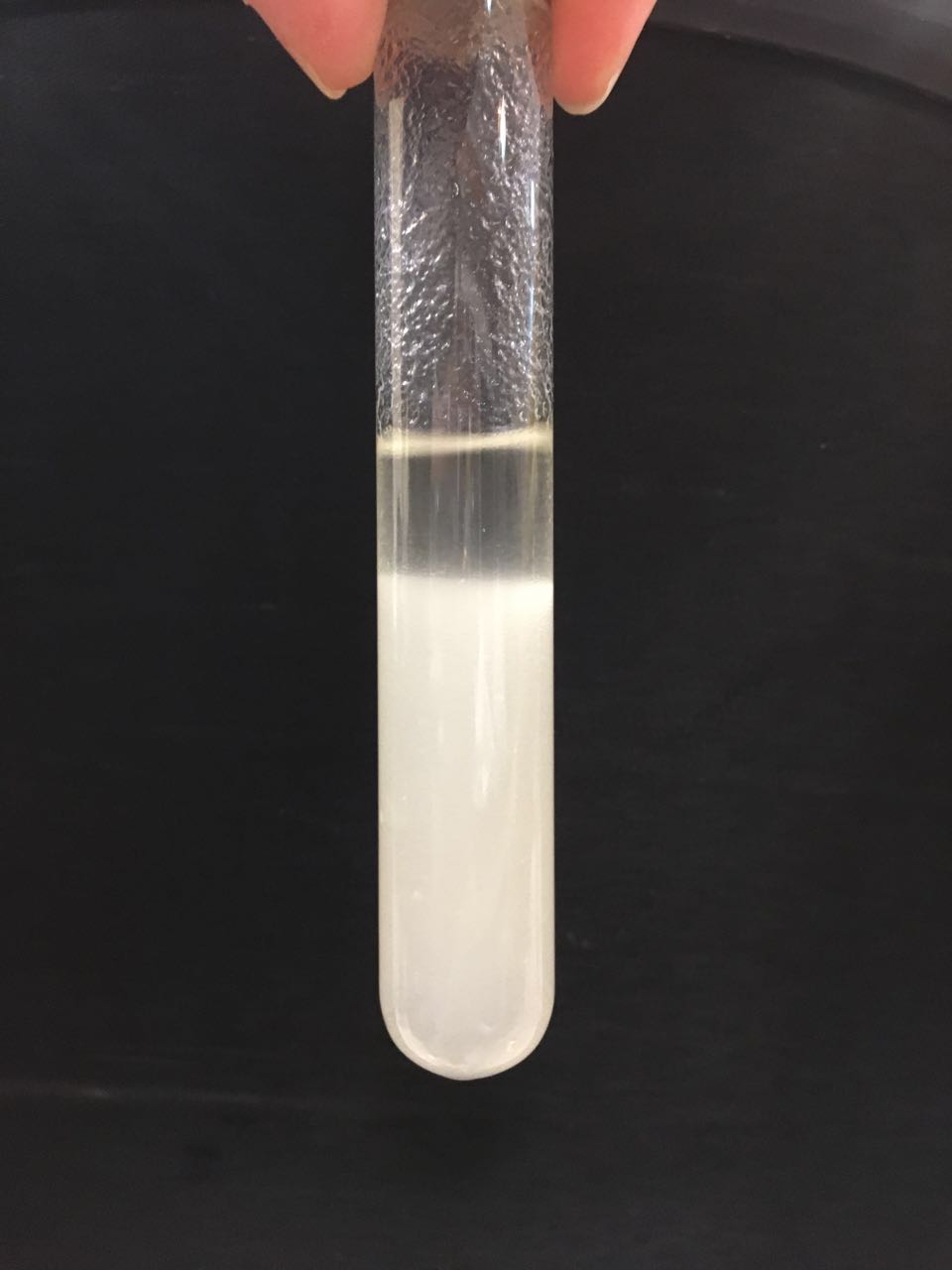 | **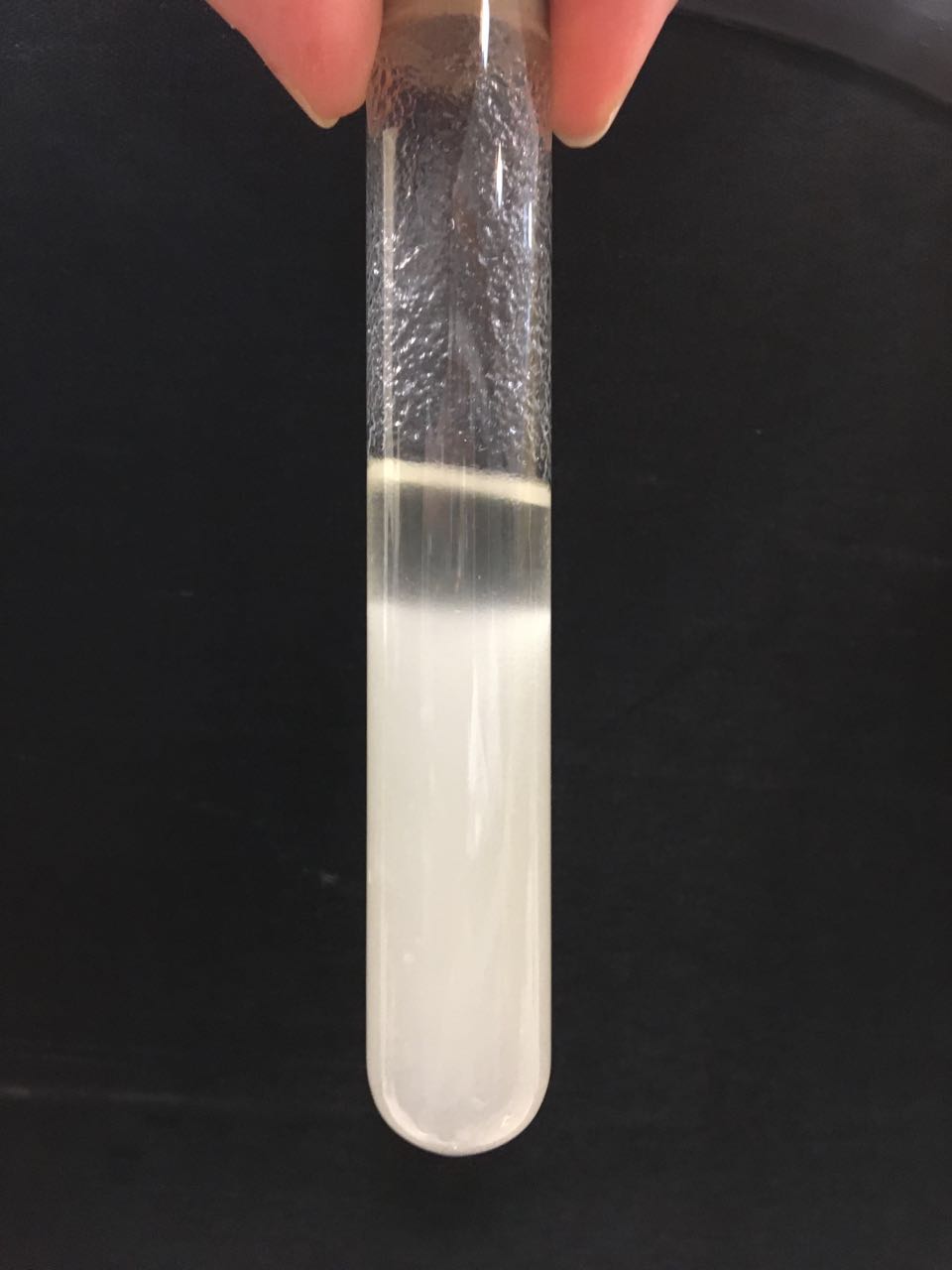** | 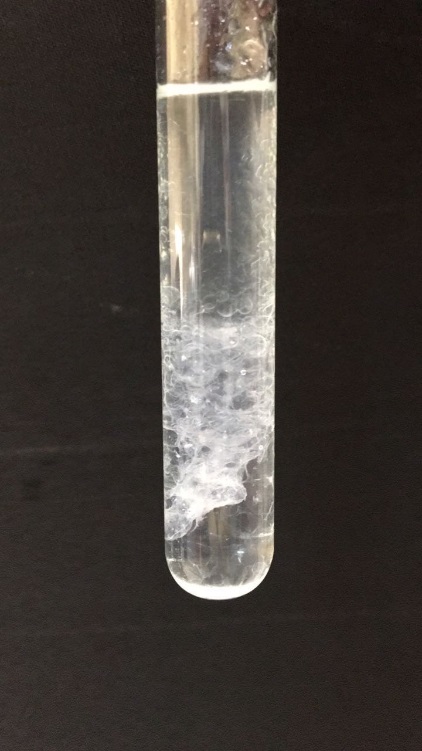 |
| --- | --- | --- |
| **A.** | **B.** | **C.** |

**Figure S2. |** Synergistic effect of the $\mathrm{Cu}^{2+}$ and nano-emulsion; during formation of the AgCl particles after adding $\mathrm{Ag}^{+}$ to the treated condensate by A) nano-emulsion (10.40 gL^-1^ oil) without any cation, B) aqueous $\mathrm{Cu}^{2+}$ solution (7.4×10^-3^ mol L^-1^), and C) nano-emulsion (10.40 gL^-1^ oil) in the presence of the $\mathrm{Cu}^{2+}$ (7.4×10^-3^ mol L^-1^) at room temperature under similar conditions.

## 3.5.SI. Optimization of the oil and the $\mathbf{Cu}^{\mathbf{2+}}$ concentration for nano-emulsion preparation

Based on the implemented semi-quantitative test, increasing the oil and/or $\mathrm{Cu}^{2+}$ concentration during the preparation of the nano-emulsion majorly promoted the extraction efficiency (Figures S3 and S4). Therefore, it was tried to decrease the $\mathrm{Cu}^{2+}$ concentration using maximum amounts of oil. This selection was related to i) decrease the probable side effect(s) of the $\mathrm{Cu}^{2+}$ residual (that would probably be remained in the NGC sample) and ii) reduce the whole price (cost) of the nano-emulsion preparation process.

As directly shown in Figures S4 and S5, the lack of any sign(s) for the AgCl formation, even, by adding very excess amount of the $\mathrm{Ag}^{+}$, revealed the absence (removal) of the $\mathrm{Cl}^{-}$ in the tested NGC. Subsequently, this issue was considered as a direct probe for directly checking the development of the extraction process. Hence, final optimum concentrations of each oil and the $\mathrm{Cu}^{2+}$ reagents were estimated to be 10.40 gL^-1^ and 7.4×10^-3^ mol L^-1^, respectively.

| 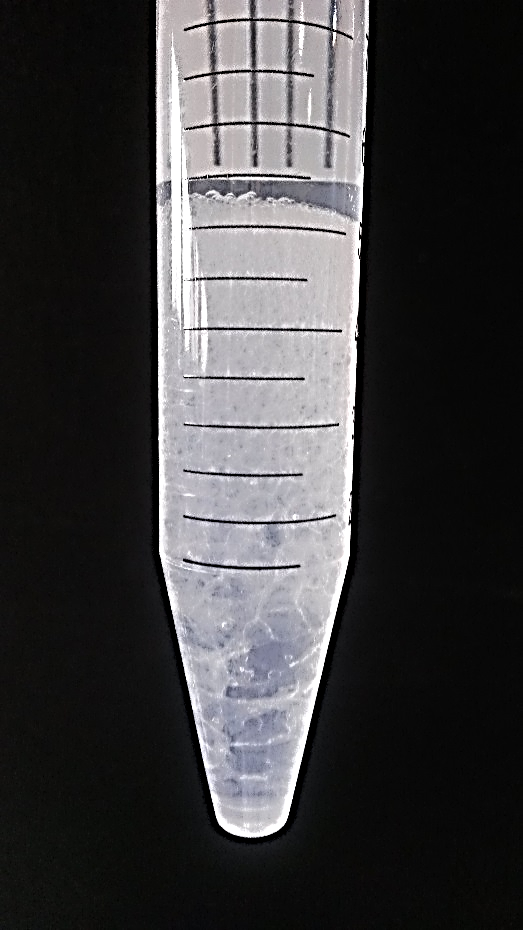 | 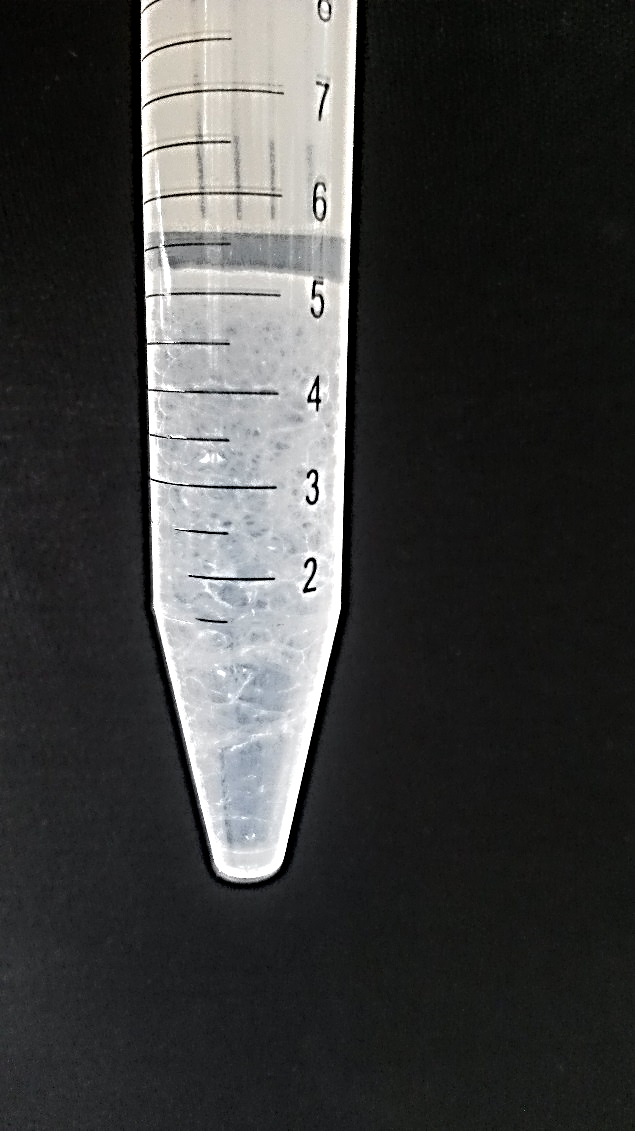 | 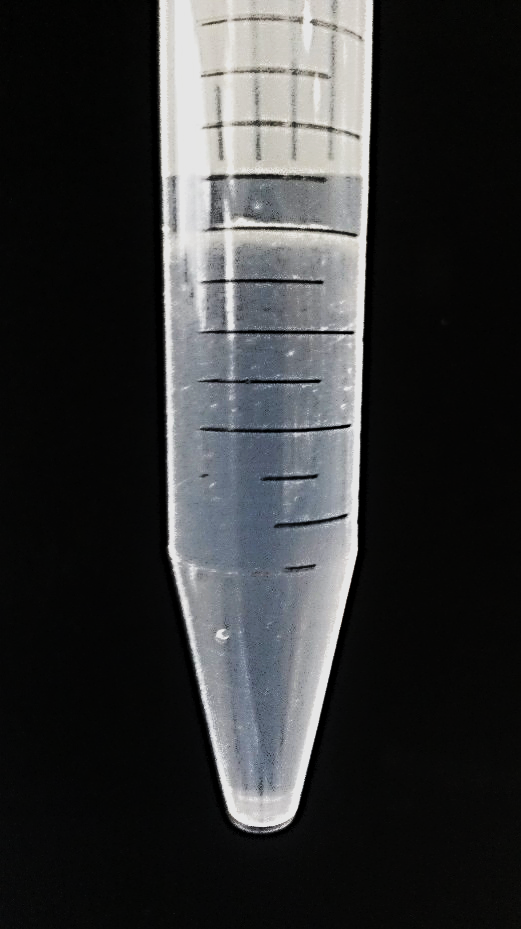 |
| --- | --- | --- |
| **A.** | **B.** | **C.** |

**Figure S3. |** Semi-quantitative tests after performing extraction by using nano-emulsion contain different oil concentration i.e. A) 2.60, B) 5.20, C) 10.40 and D) 20.80 (±0.01) g L^-1^; and the same $\mathrm{Cu}^{2+}$ concentration (7.4×10^-3^ mol L^-1^) at room temperature under similar conditions.


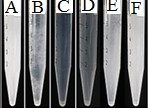

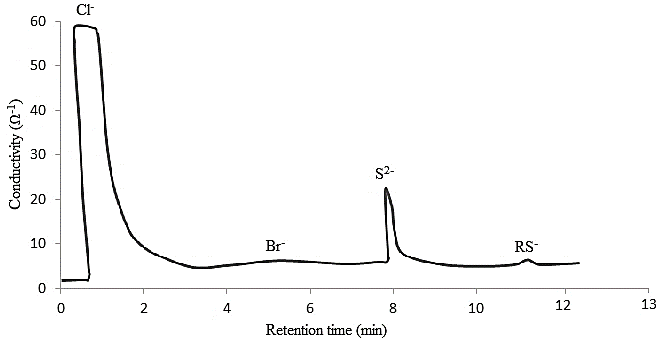


**Figure S4.** | Left) Ion-exchange chromatogram of the raw NGC and right) semi-quantitative tests after performing extraction by using nano-emulsion contain different $\mathrm{Cu}^{2+}$ concentration i.e. A) 1.0×10^-3^, B) 4.2×10^-3^, C) 7.4×10^-3^, D) 1.3×10^-2^, E) 1.6×10^-2^ and F) 2.0×10^-2^ mol L^-1^; and the same oil concentration (10.40 g L^-1^) at room temperature under similar conditions.

## 3.6.SI. Optimization the pH of the nano-emulsion for the extraction step

About this nano-emulsion, it was impossible to follow the Cl^-^ and Hg(0) removal process at the acidic condition. As this condition not only would contaminate the NGC, but also could seriously increase the corrosion characteristic; which was in disagreement with aim of this work. In addition, strong basic condition may change the matrix of the nano-emulsion, probably, due to the ligand and nucleophilic behaviors of ${OH}^{-}$, besides increase in its impurity during formation of colloidal metal hydroxides. These side effect(s) would reduce the value of the condensate. As a result, for more simplicity, as well as less deviation (unconventionality) from the natural matrix of the raw NGC sample, it was decided to focus on the neutral conditions without any necessity to add any kind(s) of buffer reagent(s). This condition therefore led to have extraction process without applying any major alteration(s) in the natural matrix of the NGC fluids.

## 3.7.SI. Different interactions between Hg(0), $\mathbf{Cu}^{\mathbf{2+}}$ and $\mathbf{Cl}^{\mathbf{-}}$

About the Hg(0) removal process, the most probably interactions were based on the $\mathrm{Cu}^{2+}$ reduction and Hg(0) oxidation in presence of the $\mathrm{Cl}^{-}$ ions during their transfer to nano-emulsion phase using the extraction step. Obviously, this process was attributed to their standard reduction potentials [4] throughout the formation of a galvanic cell according to the Equations S1-S3. These interactions would therefore result in the formation of different species such as those predicated in Equations S4-S6.

$2 Hg+2 \mathrm{Cl}^{-}+\mathrm{Cu}^{2+} \rightleftharpoons\mathrm{Hg}_{2}\mathrm{Cl}_{2(s)}+Cu$ (Eq. S1)

$\mathrm{Cu}^{2+}+2 e\rightleftharpoons\mathrm{Cu}_{(s)}$ (Eq. S2)

$\mathrm{Hg}_{2}\mathrm{Cl}_{2(s)}+ 2 e\rightleftharpoons2 Hg+ 2 \mathrm{Cl}^{-}$ (Eq. S3)

${2 [\mathrm{CuCl}_{4}]}^{2-}+Hg\rightleftharpoons{{[\mathrm{HgCl}_{4}]}^{2-}+ {2 [\mathrm{CuCl}_{2}]}^{-}}$ (Eq. S4)

${2 [\mathrm{CuCl}_{6}]}^{2-}+2 Hg\rightleftharpoons{{[{\mathrm{Hg}_{2}\mathrm{Cl}}_{6}]}^{2-}+ {2 [\mathrm{CuCl}_{2}]}^{-}}+2 \mathrm{CuCl}_{(s)}$ (Eq. S5)

$\left[ {\mathrm{Cu}_{2}\mathrm{Cl}}_{6} \right]^{2-}+ Hg\rightleftharpoons\mathrm{HgCl}_{2\left( s \right)}{+ {2 \left[ \mathrm{CuCl}_{2} \right]}^{-}}$ (Eq. S6)

It should be noted that, this suggestion was also based on the probable recommended interactions for the removal of elemental mercury from the condensate using ionic liquids [5]. However, it was proposed that, if another element such as Pb, Sn, Cd, Zn, Mn, Mg, etc. world probably be present in NGC sample, they also could be removed based on their standard reduction potentials [4] during spontaneous oxidation to their stable ionic forms.

All these probable interactions strongly revealed that, the chloride-based particles (colloids) could be suspended in the NGC matrix. Consequently, the introduced nano-emulsion had majorly probably digested almost all the forms of chloride species from the NGC matrix. Also, as there were some metals in form of non-organic compounds in emulsion form [6], the synthesized nano-emulsion had probably capable to remove them too, which mainly revealed the justification of this introduced nano-emulsion.

##

## 3.8.SI. Effect of the $\mathbf{Cu}^{\mathbf{2+}}$ concentration on the precipitation step

In this study, $\mathrm{Cu}^{2+}$ had dual roles in the precipitation step:

-1) Responsible agent for the $\mathrm{Cu}_{2}{(OH)}_{3}\mathrm{Cl}$ synthesis;

-And 2) de-emulsifying agent during total removal of oil from the nano-emulsion and transfer the aqueous phase.

Based on the results, the de-emulsifying process was not occurred without introducing any $\mathrm{Cu}^{2+}$ species, even after centrifuging for long time at high rpm speed. Consequently, it was recommended to estimate the optimum $\mathrm{Cu}^{2+}$ concertation via characterization of the $\mathrm{Cu}_{2}{(OH)}_{3}\mathrm{Cl}$ nanoparticles using the recommend techniques: Fourier transform infrared (FT-*IR*), X-ray diffraction (*XRD*), X-ray fluorescence (*XRF*) and X-ray photoelectron spectroscopy (*XPS*). This process not only led to the formation of $\mathrm{Cu}_{2}{(OH)}_{3}\mathrm{Cl}$ nanoparticles from the $\mathrm{Cl}^{-}$ ions, which were considered as bothering species inside the raw NGC, but also resulted in the re-usability of the oil to the condensate extraction cycle.

## 3.9.SI. Stability of the $\mathbf{Cu}_{\mathbf{2}}\mathbf{(OH)}_{\mathbf{3}}\mathbf{Cl}$ nanoparticles

At basicity (pH) values higher than 7.5 ± 0.1, the $\mathrm{Cu}_{2}{(OH)}_{3}\mathrm{Cl}$ particles were unstable and could be converted to ${Cu(OH)}_{2\left( S \right)}$ (Eq. S7): [7, 8].

$\mathrm{Cu}_{2}{(OH)}_{3}Cl+ \mathrm{OH}^{=}\rightleftharpoons2 {Cu(OH)}_{2(S)}+ \mathrm{Cl}^{-}$ (Eq.S7)

Therefore, pH controlling was considered as the most important factor in the workup step. Beside this, wet solid-state product was instable stable for a long time (~24 h), based on the conversion in its color from light blue to the dark blue. Also, as the solid-state product was unstable at temperatures higher than ~ 200 ^o^C, consequently, it was recommended to dry the sediment via flowing air using a fan to remove any humidity from the matrix of the $\mathrm{Cu}_{2}{(OH)}_{3}\mathrm{Cl}$ nanoparticles.

**References**

[1] M. Ergun, A.Y. Turan, Pitting potential and protection potential of carbon steel for chloride ion and the effectiveness of different inhibiting anions, Corros. Sci. 32 (1991) 1137–1142. https://doi.org/https://doi.org/10.1016/0010-938X(91)90098-A.

[2] A. Mohammadi, D. Mm, S. Mmr, Selective Speciation of Cr(III) and Cr(VI) by Micro-emulsion Using UV-Vis.Spectrophotometry, J. Text. Sci. Eng. 2016 (2016) 1–6.

[3] J. Ganchoff, Analytical Chemistry: an Introduction, Sixth Edition (Skoog, Douglas A.; West, Donald M.; Holler, F. James), J. Chem. Educ. 71 (1994) A310. https://doi.org/10.1021/ed071pA310.2.

[4] G. Milazzo, S. Caroli, R.D. Braun, Tables of Standard Electrode Potentials, J. Electrochem. Soc. 125 (1978) 261C--261C. https://doi.org/10.1149/1.2131790.

[5] M. Abai, M.P. Atkins, A. Hassan, J.D. Holbrey, Y. Kuah, P. Nockemann, A.A. Oliferenko, N. V Plechkova, S. Rafeen, A.A. Rahman, R. Ramli, S.M. Shariff, K.R. Seddon, G. Srinivasan, Y. Zou, An ionic liquid process for mercury removal from natural gas, Dalt. Trans. 44 (2015) 8617–8624. https://doi.org/10.1039/C4DT03273J.

[6] M.J. Gary, J. H.; Handwerk, G. E.; Kaiser, Petroleum refining: technology and economics., CRC press:, 2007. https://www.amazon.com/Petroleum-Refining-Technology-Economics-Fifth/dp/0849370388.

[7] Y. Jia, W.; Reitz, E.; Sun, H.; Li, B.; Zhang, H.; Lei, From Cu 2 (OH) 3 Cl to nanostructured sisal-like Cu (OH) 2 and CuO: synthesis and characterization., J. Appl. Phys. 105 (2009) 064917.

[8] K. Chen, D. Xue, Room-Temperature Chemical Transformation Route to CuO Nanowires toward High-Performance Electrode Materials, J. Phys. Chem. C. 117 (2013) 22576–22583. https://doi.org/10.1021/jp4081756.
